# The Effects of Previous Asthma and COPD on the Susceptibility to and Severity of COVID-19: A Nationwide Cohort Study in South Korea

**DOI:** 10.3390/jcm10204626

**Published:** 2021-10-09

**Authors:** Younghee Jung, Jee Hye Wee, Joo-Hee Kim, Hyo Geun Choi

**Affiliations:** 1 Division of Infectious Diseases, Department of Internal Medicine, Hallym University Sacred Heart Hospital, College of Medicine, Hallym University, Anyang 14068, Korea; jubaragiy@gmail.com; 2 Department of Otorhinolaryngology, College of Medicine, Hallym University, Anyang 14068, Korea; weejh07@hanmail.net; 3 Division of Pulmonary, Allergy and Critical Care Medicine Department of Medicine, Hallym University Sacred Heart Hospital, College of Medicine, Hallym University, Anyang 14068, Korea; luxjhee@gmail.com; 4 Department of Otorhinolaryngology-Head & Neck Surgery, College of Medicine, Hallym University, Anyang 14068, Korea; 5 Hallym Data Science Laboratory, College of Medicine, Hallym University, Anyang 14068, Korea

**Keywords:** COVID-19, COPD, asthma

## Abstract

Background: There is controversial evidence of the associations of asthma and chronic obstructive pulmonary disease (COPD) with the risk and outcomes of Coronavirus Disease 2019 (COVID-19). We aimed to evaluate the effects of asthma and COPD on the susceptibility to and severity of COVID-19. Methods: Data from a nationwide COVID-19 cohort database by the Korea National Health Insurance Corporation were utilized. A total of 4066 COVID-19 patients (1 January 2020 through 4 June 2020) were 1:4 matched with 16,264 controls with regard to age, sex, and income. Asthma and COPD were defined as diagnostic codes (ICD-10) and medication claim codes. Conditional and unconditional multivariate logistic regression were applied to analyze the susceptibility to and severity of COVID-19 associated with asthma and COPD. Results: The prevalence of mild and severe asthma/COPD did not differ between the COVID-19 and control patients in the multivariate analyses. Among the total 4066 COVID-19 patients, 343 (8.4%) had severe COVID-19, of whom 132 (3.2% of the total COVID-19 patients) died. Regarding the outcomes of COVID-19, neither mild nor severe asthma were associated with the severity or mortality of COVID-19 after adjusting for other variables. However, severe COPD was a significant risk factor for severe COVID-19 (odds ratio (OR) = 2.23, 95% confidence intervals (CI): 1.08–4.60, *p* = 0.030) and the mortality of COVID-19 in the multivariate analyses (OR = 3.06, 95% CI: 1.14–8.2, *p* = 0.026). Conclusions: In a Korean nationwide cohort, neither asthma nor COPD were associated with COVID-19, but severe COPD was associated with the severity and mortality of COVID-19.

## 1. Introduction

The COVID-19 pandemic is ongoing worldwide with global vaccination efforts. This pandemic is of particular concern for patients with chronic respiratory diseases because it is already known that respiratory virus infections are associated with more severe outcomes in these patients [1]. Moreover, a severe form of COVID-19 invades the lungs, and COVID-19 could be deadly, especially in patients with compromised lung function. Because of these concerns, several studies have tried to clarify the association between chronic lung disease and excess risk for contracting COVID-19 and severe outcomes due to COVID-19. However, the current data are conflicting about the impact of chronic respiratory diseases on the susceptibility to and severity of COVID-19 [2,3].

Early studies reported that COPD was associated with poor outcomes even though whether COPD itself was a risk factor for contracting SARS-CoV-2 was debatable [4,5,6,7]. Asthma, a generally milder respiratory disease in terms of conserved lung function, was not associated with an increased risk of SARS-CoV-2 infection in some studies [2,8], but other studies showed a positive association [9]. In addition, the association between asthma and the severity of COVID-19 was not confirmed [2,10,11]. Several meta-analyses that showed an association between asthma or COPD and the risk and consequences of COVID-19 were conducted to overcome the limitations of individual studies [3,12,13,14]. However, these meta-analyses suffer from substantial heterogeneity. In addition, most of the primary studies did not have well-matched control patients or did not adjust other variables to evaluate the effect of chronic respiratory disease on the susceptibility and outcomes of COVID-19. Moreover, large-scale nationwide epidemiological studies that investigated the relationship between asthma/COPD and COVID-19 are scarce. 

Therefore, we aimed to evaluate the association between previous asthma/COPD and the susceptibility of patients to COVID-19 in a nationwide cohort. In addition, as a secondary objective, we attempted to estimate the association between previous asthma/COPD and the severity and mortality of COVID-19.

## 2. Materials and Methods

### 2.1. Ethics

The Ethics Committee of Hallym University (22 July 2020) permitted this study. Written informed consent was waived by the Institutional Review Board. All analyses adhered to the guidelines and regulations of the Ethics Committee of Hallym University. 

### 2.2. Study Population and Participant Selection

We used Korea National Health Insurance Database Coronavirus disease 2019 (NHID-COVID DB) medical claim code data from 2015 through 2020. NHID-COVID DB provided the data of individuals who underwent SARS-CoV-2 testing using real-time reverse transcriptase–PCR assay of nasal or pharyngeal swabs in accordance with the WHO guidelines, and control patients were proportionally sampled from the database of Korean National Health Insurance stratifying by age and sex. 

**Figure 1:**
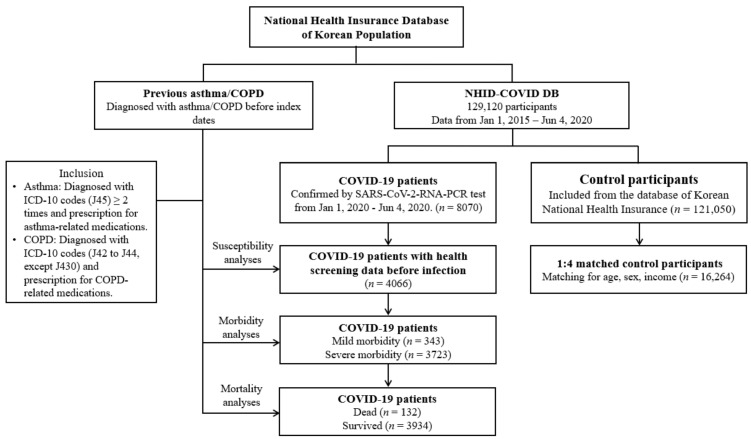
A schematic illustration of the participant selection process that was used in the present Scheme 129. 4066 COVID-19 patients were matched with 16,264 control participants for age, sex, and income. Abbreviation: ICD-10, International classification of disease-10; COPD, Chronic obstructive pulmonary disease.

Patients who were confirmed from 1 January 2020 through 4 June 2020 were included, and all the patients were followed until discharge from the hospital or residential center or death during admission (*n* = 8070). The control patients (*n* = 121,050) were extracted at fifteen times the number of COVID-19 patients from the National Health Insurance Sharing Service (NHISS) database (https://nhiss.nhis.or.kr/ accessed on 15 January 2021). The control group did not include COVID-19 patients. We matched COVID-19 patients who had national health screening data prior to infection (*n* = 4066) with control patients at a 1:4 ratio in terms of age, sex, and income. Among control patients, we excluded patients with a lack of income records (*n* = 2136). To avoid selection bias, control patients were selected randomly using clustered sampling. The index date was defined as the date of confirmation of COVID-19. Each index date of control patients was selected randomly from 1 January 2020 to 4 June 2020. As a consequence, 4066 COVID-19 patients were matched 1:4 with 16,264 control patients. The previous prevalence of asthma and COPD was analyzed in COVID-19 patients and control patients. The prevalence of asthma and COPD was analyzed for mild (*n* = 3723) and severe (*n* = 343) COVID-19, then for death (*n* = 132), and survival (*n* = 3934) among the severe COVID-19 patients (Figure 1).

### 2.3. Exposure (Asthma)

Asthma was defined as patients who were treated for asthma (ICD-10: J45) or status asthmaticus (J46) ≥ 2 times with asthma-related medications, including inhaled corticosteroids (ICSs) or ICSs combined with long-acting β2-agonists (LABAs), oral leukotriene antagonists (LTRAs), short-acting β2-agonists (SABAs), systemic LABAs, xanthine derivatives, or systemic corticosteroids. Severe asthma was defined as using one of the following medications within two years: (1) ICSs/LABAs + long-acting muscarinic antagonists (LAMAs), (2) ICSs/LABAs + LTRAs, (3) ICSs/LABAs + xanthine, and (4) corticosteroids for over 90 days. Otherwise, it was defined as mild.

### 2.4. Exposure (COPD)

Chronic obstructive pulmonary disease (COPD) was defined by unspecified chronic bronchitis (J42), emphysema (J43), J44 (other COPD (J44) except MacLeod syndrome (J430)) and the administration of LAMAs, LABAs, ICSs combined with LABAs, short-acting muscarinic antagonists (SAMAs), SABAs, methylxanthine, PDE4 inhibitors, and systemic beta agonists. Severe COPD was defined as a history of using systemic corticosteroids within the previous two years. Otherwise, it was defined as mild.

### 2.5. Outcome (COVID-19)

Laboratory confirmation of a SARS-CoV-2 infection was defined as the primary outcome.

### 2.6. Secondary Outcome (Severity and Mortality)

The secondary outcomes were severity and mortality in COVID-19 patients. Severity was defined as severe disease compared to mild disease. Severe disease was defined by admission to the intensive care unit, invasive ventilation, extracorporeal membrane oxygenation, and death. 

### 2.7. Covariates

Age groups were divided into 10-year intervals: 20–29, 30–39…, and 80+ years old (total of 7 age groups). Income groups were classified into 3 classes (low income, middle income, and high income). Missing income (*n* = 78 (0.38%)) was replaced by middle income groups. Tobacco smoking, alcohol consumption, and obesity status using BMI (body mass index, kg/m^2^) were categorized as described in our previous study [15]. The records of systolic blood pressure (SBP, mmHg), diastolic blood pressure (DBP, mmHg), fasting blood glucose levels (mg/dL), and total cholesterol levels (mg/dL) were used.

The Charlson comorbidity index (CCI) has been used widely to measure disease burden using 17 comorbidities as the continuous variables (0 (no comorbidities) through 29 (multiple comorbidities)) [16]. In our study, we excluded respiratory diseases from CCI score.

Regarding asthma and COPD, hypertension (ICD-10 codes: I10 and I15, treated ≥ 2 times), steroid prescriptions, and NSAID (non-steroidal anti-inflammatory drug) prescriptions were additionally assigned covariates.

### 2.8. Statistical Analyses

The general characteristics between the COVID-19 and control groups and between the severe and mild COVID-19 groups were compared using the chi-square test or Fisher’s exact test for categorical variables and the independent *t*-test for continuous variables.

To estimate the susceptibility of COVID-19 patients compared to control patients, odds ratios (ORs) with 95% confidence intervals (CIs) for asthma and COPD were calculated using crude analyses (simple model) for Model 1 (adjusted for obesity status, smoking, alcohol consumption, systolic blood pressure, diastolic blood pressure, fasting blood glucose levels, total cholesterol levels, CCI scores, number of NSAIDs used, number of steroids used, and hypertension history) and Model 2 (Model 1 plus asthma or COPD) with conditional logistic regression. In these analyses, age, sex, and income were stratified. To estimate the severity and mortality in COVID-19 patients by asthma and COPD, unconditional logistic regression was used for the unmatched analyses. In addition, subgroup analyses were performed for all covariates.

For the statistical analyses, SAS version 9.4 (SAS Institute Inc., Cary, NC, USA) was used. We performed two-tailed analyses, and significance was defined as *p* values less than 0.05.

## 3. Results

### 3.1. Susceptibility to COVID-19

The general characteristics of the patients are described in Table 1. A total of 4066 COVID-19 patients were paired with 16,264 control patients with a 1:4 match. The baseline characteristics of the COVID-19 and control patients were well balanced according to age, sex, and income (all *p* = 1.0) Non-smokers were more common in the control groups, but alcohol consumption was more frequent in the COVID-19 groups (*p* < 0.001). High systolic blood pressure was more frequently presented in the control groups than in the COVID-19 groups (*p* < 0.001). Obese and overweight patients were more commonly encountered in the COVID-19 groups (*p* = 0.001). The prevalence rates of mild and severe asthma were 7.9% (322/4066) and 1.0% (40/4066), respectively, in the COVID-19 patients. Among the controls, the prevalence rates of mild and severe asthma were 7.1% (1616/16,264) and 0.5% (136/16,264), respectively. The prevalence of mild and severe asthma was not different between the two groups (*p* = 0.147). In the subgroup analysis with men (*n* = 7630), mild asthma was more common in the COVID-19 patients than in the control patients (OR = 1.30, 95% CI= 1.01–1.66, *p* = 0.039) (Table S1). A subgroup analysis with smoking history or current smoking showed that mild asthma was associated with COVID-19 (OR = 1.36, 95% CI = 1.03–1.79, *p* = 0.031). After adjusting for other variables, including COPD, asthma was not associated with COVID-19 regardless of the severity of the asthma (*p* = 0.27 for mild asthma and *p* = 0.51 for severe asthma) (Table 2).

The prevalence rates of mild and severe COPD were 2.5% (101/4066) and 1.6% (63/4066), respectively, in the COVID-19 patients. Among the controls, the prevalence rates of mild and severe COPD were 2.2% (358/16,264) and 1.1% (180/16,264), respectively. The presence and severity of COPD were significantly different between the patients with COVID-19 and controls in the crude analyses (*p* = 0.037) (Table 1). In particular, severe COPD was more common in the COVID-19 patients than in the controls (OR = 1.41, 95% CI = 1.06¬–1.89, *p* = 0.019), but statistical significance was not reached in the multivariate analyses (OR = 1.30, 95% CI = 0.96¬–1.76, *p* = 0.095). In the subgroup analyses according to body weight, among the patients with normal weight (*n* = 7819), severe COPD was significantly associated with COVID-19 in the multivariate analyses (OR = 1.18, 95% CI = 1.17–3.03, *p* = 0.010). In patients who were underweight (*n* = 700), mild COPD was associated with COVID-19 in the adjusted analyses (OR = 12.56, 95% CI = 3.62–43.59, *p* < 0.001) (Table S1).

### 3.2. COVID-19 Severity and Mortality 

The characteristics of mild and severe COVID-19 are compared in Table 1. Among the 4066 COVID-19 patients, 343 patients (8.4%) were classified as having severe disease; of these, 132 patients (3.2% of the total COVID-19 patients) died. The distributions of age, sex, income, obesity status, smoking status, and systolic blood pressure were different between patients with mild and severe COVID-19 (all *p* <0.001). A higher CCI score was more common in patients with severe COVID-19, and the frequency of steroid use was higher in patients with severe COVID-19 than in patients with the mild disease (*p* < 0.001). There were differences in asthma (*p* = 0.004) and COPD status (*p* < 0.001) between the two groups in the univariate analyses. In the multivariate analyses, asthma was not associated with the severity of COVID-19 (OR = 1.08, 95% CI = 0.72–1.63, *p* = 0.71 in mild asthma and OR = 0.50, 95% CI = 0.14–1.80, *p* = 0.29 for severe asthma) (Table 3). For COPD, severe COPD was significantly associated with a severe outcome of COVID-19 in the adjusted analyses (OR = 2.23, 95% CI = 1.05–4.36, *p* = 0.030) (Table 3). In contrast with past and current smokers, the subgroup analyses with non-smokers showed that severe COPD was a significant risk factor for severe COVID-19 (OR = 2.46, 95% CI = 1.06–5.70, *p* = 0.037) (Table S2).

In the mortality analyses, 132 patients (3.4% of the total COVID-19 patients) died. Mild and severe asthma were associated with mortality in the crude analyses, but, in the multivariate analyses, asthma was not significantly associated with mortality (*p* = 0.60 for mild asthma and *p* = 0.67 for severe asthma) (Table 4). Both mild and severe COPD were associated with mortality in the crude analyses, but, after adjusting for other variables, only severe COPD remained significant (OR = 3.06, 95% CI =1.14¬– 8.20, *p* = 0.026) (Table 4). In contrast with past or current smokers, the subgroup analyses with non-smokers showed that severe COPD was a significant risk factor for mortality (OR = 3.53, 95% CI =1.06–11.76, *p* = 0.04) (Table S3).

## 4. Discussion

This study suggests that asthma and COPD were not risk factors for contracting COVID-19. For COVID-19 outcomes, severe COPD was significantly associated with the severity and mortality of COVID-19 in contrast with asthma. To our knowledge, this is the first nationwide cohort study to evaluate the associations of asthma and COPD with COVID-19 and their impacts on the outcome of COVID-19. 

There is great variability in the prevalence of asthma in COVID-19-infected patients according to regional area [3]. Studies in United States showed the pooled prevalence of asthma in COVID-19 was 11.0% (95% CI = 9.8–12.3%), while Europe reported 7.6% (95% CI, 6.0–9.4%). In China, the pooled prevalence of asthma was much lower (1.9%, 95% CI 0.4–4.4%) [17]. Several studies, including meta-analyses, attempted to elucidate the increased risk of COVID-19 among asthmatic patients, but the studies did not demonstrate a clear association between asthma and COVID-19 [2,3,17,18]. In addition, study design also influences the results of the association between asthma and COVID-19. Simple comparisons of the prevalence of asthma in COVID-19 patients with a historical control population is not accurate for elucidating the associations between asthma and COVID-19. Such designs did not take into account other confounding variables and changing prevalence over time [17]. Most of the comparative studies used a test-negative design to assess the risk of asthmatic patients acquiring COVID-19. In other words, people who tested negative for COVID-19 served as controls and were compared to those who tested positive for COVID-19. Such a design by nature leads to selection bias because people who tested negative are not representative of the general population [19]. Patients with asthma, due to their respiratory symptoms that are not differentiated from the symptoms of COVID-19, tend to seek COVID-19 tests more readily than the general population. Consequently, the rate of asthma was reported to be higher among people who tested negative (10.2%; 95% CI, 7.5–13.3%) than among those who tested positive (7.8%; 95% CI, 5.1–11.1%) [18], which might confound the real effect of asthma on the development of COVID-19. For example, a nationwide study in Israel reported a negative association of asthma and COVID-19 in their test-negative study design [8]. In contrast, in the UK Biobank cohort, the researchers compared the prevalence of asthma among COVID-19 patients with the remaining members of their large cohort, who were not tested for COVID-19, and the prevalence of asthma was higher in those who tested positive than in the remaining population [20]. In our study, we selected a well-matched control group from the database of the Korean National Health Insurance regardless of history of COVID-19 testing and did not see any significant difference in the prevalence of either mild or severe asthma between the control and COVID-19 patients.

The prevalence of COPD among patients with COVID-19 revealed a wide-range, and most of the studies concentrated on hospitalized patients. Only limited data exist on the association between COPD and COVID-19 [21,22,23,24]. Theoretically, patients with COPD may have an increased risk for COVID-19 based on in vitro data that revealed ACE2 mRNA expression is increased in patients with COPD [25]. However, current data on the susceptibility to COVID-19 among COPD patients are discrepant [21,22,24,26]. In our study, severe COPD was more common in the patients with COVID-19 than in the controls (1.5% vs. 1.1%, *p* = 0.019), but, after adjusting for other comorbidities, the difference was not significant (*p* = 0.095). There are some possible explanations for this result. First, patients with COPD might have a very limited chance of contracting COVID-19 due to self-quarantining because of a fear of severe illness. Second, COPD underdiagnosis is a well-known issue [27], so COPD as an exposure variable might be measured inaccurately and underestimated as a risk factor for contracting COVID-19. No current evidence, including ours, provides definitive conclusions demonstrating that patients with COPD have an increased risk of acquiring COVID-19. 

In terms of COVID-19 outcomes, asthma and COPD had different behaviors in the present study. Neither mild nor severe asthma were associated with the severity or mortality of COVID-19, which is in line with previous studies [28,29,30]. The meta-analyses did not provide clear evidence of an increased risk of COVID-19-related hospitalization, severity, or mortality among people with asthma [18,31]. In contrast, a few studies have shown that asthma is associated with unfavorable outcomes of COVID-19 [9,32]. Interestingly, in both studies, non-allergic asthma was more related to severe COVID-19, along with data from the UK Biobank community cohort [18]. We could not classify asthma phenotypes, but there could be a possibility that different asthma types may have a differential impact on the severity of COVID-19. Further studies are needed to confirm this. 

In contrast with asthma, severe COPD was associated with severe COVID-19 in our study. In the mortality analyses, COPD was independently associated with death (OR = 3.06, 95% CI = 1.14–8.2, *p* = 0.026). Our findings correspond to previous studies [29,33], while other studies did not find an association between COPD and in-hospital mortality [34,35]. However, most of the studies, including systematic reviews, only compared the crude rate of COPD between mild or severe COVID-19 disease and did not adjust for other risk factors [12,14]. Patients with COPD frequently have chronic diseases, such as cardiovascular disease, and are older, which are factors known to be associated with unfavorable outcomes of COVID-19. Therefore, adjusting for other confounding variables is important to demonstrate that COPD is an independent risk factor for poor outcomes of COVID-19. In our study, we adjusted for other variables, including smoking status, which is closely related to COPD and is also a risk factor for poor outcomes of COVID-19 [36]. We examined smoking status using the Korea National Health screening data, and further analyzed smoking as a covariable, while most of the previous studies did not consider smoking status. 

Similar to our data, the different impacts of previous asthma and COPD were reported in other studies [12,13,14,37]. There are possible reasons for the results. First, poor lung function reservoirs in patients with COPD may easily precipitate respiratory failure compared to people with asthma. In addition, comorbidities, such as older age and cardiovascular disease, are more commonly present among patients with COPD and might influence the unfavorable outcomes of COVID-19 even after adjusting for these variables. Moreover, a recent in vitro study showed that ACE2 protein expression, which is responsible for the entry of SARS-CoV-2, was increased in the lower airways in patients with COPD but decreased in those with asthma, which needs to be confirmed in further research [37]. There is also the possibility of different outcomes for patients with asthma-COPD overlap syndrome and the co-occurrence of COVID-19, which needs to be analyzed in future studies. 

Our study has several strengths. This is the first nationwide study to evaluate asthma and COPD and COVID-19 risks and outcomes. Second, the outcomes of COVID-19 were accurately measured. In Korea, since early in the pandemic, there has not been a shortage of COVID-19 tests, so even asymptomatic patients could freely be tested by PCR with contact tracing or voluntary testing. Consequently, undiagnosed COIVD-19 infections seem to be very rare according to a seroprevalence study in Korea [38]. In addition, all the COVID-19 patients stayed in residential treatment centers or hospitals until recovery or death and were treated without charge. Therefore, the possible confounding effect of treatment on the outcomes could be minimized. In terms of the study design, our control group was selected from the general population, not from the test-negative population, and was more representative of the general population and less biased than in previous test-negative control studies. The results of our population-based study are generally consistent with previous studies and provide reassurance for patients with asthma and warnings for patients with severe COPD with more reliable evidence. 

This study also has limitations. First, we could not apply international guidelines in the definition of asthma and COPD and their severity because this is a population-based study using health claim data and diagnostic codes, and we could not access individual medical records like in other nationwide studies. Second, we did not investigate the effect of specific medications on the susceptibility to COVID-19 or the severity of COVID-19. Third, South Korea maintained strict infection control at the beginning of the pandemic: social distancing and universal mask-wearing have been expected nationwide, which exceedingly decreased the chances of exposure to COVID-19. This situation might have resulted in not being able to analyze a significant number of patient differences between the groups in analyzing the impact of asthma. Our data should be interpreted with caution in countries where different infection control measures have been implemented

## 5. Conclusions

In conclusion, we did not find associations of asthma and COPD with the risk of COVID-19. We demonstrated that severe COPD was associated with the severity and mortality of COVID-19; however, no association between asthma and COVID-19-related outcomes was identified. 

## Figures and Tables

**Table 1:** General characteristics of participants.

Characteristics	Total Participants			COVID-19 Participants		
	COVID-19	Control	*p*-Value	Severe Disease	Mild Disease	*p*-Value
Total number (n, %)	4066 (100.0)	16,264 (100.0)		343 (100.0)	3723 (100.0)	
Age (years old) (n, %)			1000			<0.001 *
20–29	198 (4.9)	792 (4.9)		7 (2.0)	191 (5.1)	
30–39	366 (9.0)	1464 (9.0)		13 (3.8)	353 (9.5)	
40–49	710 (17.5)	2840 (17.5)		21 (6.1)	689 (18.5)	
50–59	1212 (29.8)	4848 (29.8)		51 (14.9)	1161 (31.2)	
60–69	945 (23.2)	3780 (23.2)		92 (26.8)	853 (22.9)	
70–79	436 (10.7)	1744 (10.7)		80 (23.3)	356 (9.6)	
80+	199 (4.9)	796 (4.9)		79 (23.0)	120 (3.2)	
Sex (n, %)			1000			<0.001 *
Male	1526 (37.5)	6104 (37.5)		204 (59.5)	1322 (35.5)	
Female	2540 (62.5)	10,160 (62.5)		139 (40.5)	2401 (64.5)	
Income (n, %)			1000			<0.001 *
1 (low)	1472 (36.2)	5888 (36.2)		88 (25.7)	1384 (37.2)	
2	1249 (30.7)	4996 (30.7)		110 (32.1)	1139 (30.6)	
3 (high)	1345 (33.1)	5380 (33.1)		145 (42.3)	1200 (32.2)	
Obesity			0.001 *			<0.001 *
Underweight	129 (3.2)	571 (3.5)		5 (1.5)	124 (3.3)	
Normal	1458 (35.9)	6361 (39.1)		103 (30.0)	1355 (36.4)	
Overweight	1010 (24.8)	3858 (23.7)		70 (20.4)	940 (25.3)	
Obese	1469 (36.1)	5474 (33.7)		165 (48.1)	1304 (35.0)	
Smoking status (n, %)			<0.001 *			<0.001 *
Non-smoker	3181 (78.2)	11,572 (71.2)		233 (67.9)	2948 (79.2)	
Past smoker	603 (14.8)	2085 (12.8)		92 (26.8)	511 (13.7)	
Current smoker	282 (6.9)	2607 (16.0)		18 (5.3)	264 (7.1)	
Alcohol consumption (n, %)			<0.001 *			0.333
<1 time a week	2968 (73.0)	10,497 (64.5)		258 (75.2)	2710 (72.8)	
≥1 time a week	1098 (27.0)	5767 (35.5)		85 (24.8)	1013 (27.2)	
Systolic blood pressure (n, %)			<0.001 *			<0.001 *
<120 mmHg	1816 (44.7)	6756 (41.5)		104 (30.3)	1712 (46.0)	
120–139 mmHg	1809 (44.5)	7484 (46.0)		167 (48.7)	1642 (44.1)	
≥140 mmHg	441 (10.9)	2024 (12.4)		72 (21.0)	369 (9.9)	
Diastolic blood pressure (n, %)			0.010 *			0.061
<80 mmHg	2645 (65.1)	10,164 (62.5)		204 (59.5)	2441 (65.6)	
80–89 mmHg	1114 (27.4)	4767 (29.3)		106 (30.9)	1008 (27.1)	
≥90 mmHg	307 (7.6)	1333 (8.2)		33 (9.6)	274 (7.4)	
Fasting blood glucose (n, %)			0.103			<0.001 *
<100 mg/dL	2522 (62.0)	10,284 (63.2)		153 (44.6)	2369 (63.6)	
100–125 mg/dL	1170 (28.8)	4644 (28.6)		123 (35.9)	1047 (28.1)	
≥126 mg/dL	374 (9.2)	1336 (8.2)		67 (19.5)	307 (8.3)	
Total cholesterol (n, %)			0.249			0.023 *
<200 mg/dL	2325 (57.2)	9068 (55.8)		220 (64.1)	2105 (56.5)	
200–239 mg/dL	1246 (30.6)	5123 (31.5)		90 (26.2)	1156 (31.1)	
≥240 mg/dL	495 (12.2)	2073 (12.8)		33 (9.6)	462 (12.4)	
CCI score (n, %)			<0.001 *			<0.001 *
0	3089 (76.0)	14,609 (89.8)		164 (47.8)	2925 (78.6)	
1	588 (14.5)	866 (5.3)		91 (26.5)	497 (13.4)	
≥2	389 (9.6)	789 (4.9)		88 (25.7)	301 (8.1)	
The number of NSAID use (mean, SD)	10.13 (12.21)	9.60 (13.03)	0.015 ^†^	14.53 (22.36)	9.72 (10.72)	<0.001 ^†^
The number of steroid use (mean, SD)	4.51 (7.23)	4.45 (7.33)	0.664	7.05 (10.89)	4.28 (6.75)	<0.001 ^†^
Hypertension (n, %)	1188 (29.2)	4601 (28.3)	0.241	178 (51.9)	1010 (27.1)	<0.001 *
Asthma (n, %)			0.147			0.004 *
Non-asthma	3704 (91.1)	14,967 (92.3)		296 (86.3)	3408 (91.5)	
Mild	322 (7.9)	1161 (7.1)		43 (12.5)	279 (7.5)	
Severe	40 (1.0)	136 (0.8)		4 (1.2)	36 (1.0)	
COPD (n, %)			0.037 *			<0.001 *
Non-COPD	3902 (96.0)	15,726 (96.7)		313 (91.3)	3589 (96.4)	
Mild	101 (2.5)	358 (2.2)		15 (4.4)	86 (2.3)	
Severe	63 (1.6)	180 (1.1)		15 (4.4)	48 (1.3)	

Abbreviations: CCI, Charlson comorbidity index; Coronavirus Disease 2019; COPD, Chronic Obstruction Pulmonary Disease.* Chi-square or Fisher’s exact test. Significance at *p* < 0.05. ^†^ Independent *t*-test. Significance at *p* < 0.05.

**Table 2:** Crude and adjusted odds ratios of asthma and COPD for COVID-19 in total participants.

Characteristics	COVID-19	Control	ORs (95% Confidence Interval) for COVID-19					
	(Exposure/ Total, %)	(Exposure/ Total, %)	Crude ^†^	*p*-Value	Model 1 ^†,‡^	*p*-Value	Model 2 ^†,§^	*p*-Value
**Asthma**								
Non-asthma	3704/4066 (91.1%)	14,967/16,264 (92.0%)	1		1		1	
Mild-asthma	322/4066 (7.9%)	1161/16,264 (7.1%)	1.12 (0.99–1.28)	0.080	1.10 (0.96–1.25)	0.175	1.08 (0.94–1.24)	0.273
Severe-asthma	40/4066 (1.0%)	136/16,264 (0.8%)	1.19 (0.84–1.70)	0.336	1.19 (0.82–1.71)	0.357	1.13 (0.78–1.64)	0.508
**COPD**								
Non-COPD	3902/4066 (96.0%)	15,726/16,264 (96.7%)	1		1		1	
Mild-COPD	101/4066 (2.5%)	358/16,264 (2.2%)	1.14 (0.91–1.43)	0.260	1.12 (0.89–1.42)	0.331	1.09 (0.86–1.39)	0.455
Severe-COPD	63/4066 (1.5%)	180/16,264 (1.1%)	1.41 (1.06–1.89)	0.019 *	1.33 (0.98–1.80)	0.064	1.30 (0.96–1.76)	0.095

Abbreviations: CCI, Charlson comorbidity index; Coronavirus Disease 2019; COPD, Chronic Obstruction Pulmonary Disease; OR, odds ratio.* Conditional logistic regression model, Significance at *p* < 0.05. ^†^ Stratified model for age, sex, and income. ^‡^ Model 1 was adjusted for obesity, smoking, alcohol consumption, systolic blood pressure, diastolic blood pressure, fasting blood glucose, total cholesterol, CCI scores, number of NSAIDs used, number of steroids used, and hypertension. ^§^ Model 2 was adjusted for model 1 plus asthma and COPD.

**Table 3:** Crude and adjusted odds ratios of asthma and COPD for morbidity in COVID-19 participants.

Characteristics	Severe Participants	Mild Participants	ORs (95% Confidence Interval) for Morbidity					
	(Exposure/Total, %)	(Exposure/Total, %)	Crude	*p*-Value	Model ^1,^^†^	*p*-Value	Model 2 ^‡^	*p*-Value
**Asthma**								
Non-asthma	296/343 (86.3%)	3408/3723 (91.5%)	1		1		1	
Mild-asthma	43/343 (12.5%)	279/3723 (7.5%)	1.78 (1.26–2.50)	0.001 *	1.13 (0.76–1.68)	0.533	1.08 (0.72–1.63)	0.709
Severe-asthma	4/343 (1.2%)	36/3723 (1.0%)	1.28 (0.45–3.62)	0.643	0.58 (0.17–2.02)	0.393	0.50 (0.14–1.80)	0.288
**COPD**								
Non-COPD	313/343 (91.3%)	3589/3723 (96.4%)	1		1		1	
Mild-COPD	15/343 (4.4%)	86/3723 (2.3%)	2.00 (1.14–3.50)	0.015	0.95 (0.49–1.83)	0.874	0.99 (0.51–1.95)	0.980
Severe-COPD	15/343 (4.4%)	48/3723 (1.3%)	3.58 (1.98–6.47)	<0.001 *	2.14 (1.05–4.36)	0.035 *	2.23 (1.08–4.60)	0.030 *

Abbreviations: Coronavirus Disease 2019; COPD, Chronic Obstruction Pulmonary Disease; OR, odds ratio.* Unconditional logistic regression model, Significance at *p* < 0.05. ^†^ Model 1 was adjusted for age, sex, income, obesity, smoking, alcohol consumption, systolic blood pressure, diastolic blood pressure, fasting blood glucose, total cholesterol, CCI scores, number of NSAIDs used, number of steroids used, and hypertension. ^‡^ Model 2 was adjusted for model 1 plus asthma or COPD.

**Table 4:** Crude and adjusted odds ratios of asthma and COPD for mortality in COVID-19 participants.

Characteristics	Dead Participants	Survived Participants	ORs (95% Confidence Interval) for Mortality					
	(Exposure/Total, %)	(Exposure/Total, %)	Crude	*p*-Value	Model 1 ^†^	*p*-Value	Model 2 ^‡^	*p*-Value
**Asthma**								
Non-asthma	108/132 (81.8%)	3596/3934 (91.4%)	1		1		1	
Mild-asthma	20/132 (15.2%)	302/3934 (7.7%)	2.21 (1.35–3.60)	0.002 *	0.96 (0.52–1.74)	0.880	0.85 (0.45–1.60)	0.605
Severe-asthma	4/132 (3.0%)	36/3934 (0.9%)	3.70 (1.29–10.58)	0.015 *	1.03 (0.22–4.75)	0.972	0.70 (0.13–3.68)	0.672
**COPD**								
Non-COPD	113/132 (85.6%)	3789/3934 (96.3%)	1		1		1	
Mild-COPD	9/132 (6.8%)	92/3934 (2.3%)	3.28 (1.61–6.67)	0.001 *	0.99 (0.40–2.49)	0.989	1.10 (0.42–2.89)	0.851
Severe-COPD	10/132 (7.6%)	53/3934 (1.3%)	6.33 (3.14–12.76)	<0.001 *	2.79 (1.09–7.17)	0.033*	3.06 (1.14–8.20)	0.026 *

Abbreviations: Coronavirus Disease 2019; COPD, Chronic Obstruction Pulmonary Disease; OR, odds ratio.* Unconditional logistic regression model, Significance at *p* < 0.05. ^†^ Model 1 was adjusted for age, sex, income, obesity, smoking, alcohol consumption, systolic blood pressure, diastolic blood pressure, fasting blood glucose, total cholesterol, CCI scores, number of NSAIDs used, number of steroids used and hypertension. ^‡^ Model 2 was adjusted for model 1 plus asthma or COPD.

## Data Availability

Releasing of the data by the researcher is not legally permitted. All data are available from the database of the Korea Center for Disease Control and Prevention. The Korea Center for Disease Control and Prevention allows data access, at a particular cost, for any researcher who promises to follow the research ethics. The data of this article can be downloaded from the website after agreeing to follow the research ethics.

## Supplementary Materials

The following are available online at https://www.mdpi.com/article/10.3390/jcm10204626/s1, Table S1: Stratified subgroup analyses of crude and adjusted odds ratios of asthma and COPD for COVID-19 in total participants by covariates; Table S2: Subgroup analyses of crude and adjusted odds ratios of asthma and COPD for morbidity in COVID-19 participants by covariates; Table S3; Subgroup analyses of crude and adjusted odds ratios of asthma and COPD for mortality in COVID-19 participants by covariates.
