# Supplementary material for: The Effects of Previous Asthma and COPD on the Susceptibility to and Severity of COVID-19: A Nationwide Cohort Study in South Korea
Source: J Clin Med. 2021 Oct 9;10(20):4626. doi: 10.3390/jcm10204626 (PMC8541474; doi:10.3390/jcm10204626)
Supplement: Supplementary file 1 [file jcm-10-04626-s001.zip › jcm-1382001-supplementary.pdf]

Table S1. Stratified subgroup analyses of crude and adjusted odds ratios of asthma and COPD for COVID-19 infection in total participants by covariates

Characteristics	COVID-19 (exposure/total, %)	Control (exposure/total, %)	ORs (95% confidence interval) for COVID-19					
			Crude†	P-value	Model 1†‡	P-value	Model 2†§	P-value
**Age < 60 years old**	**n = 2486**	**n = 9944**						
**Asthma**								
Non-asthma	2,317/2,486 (93.2%)	9,363/9,944 (94.2%)	1		1		1	
Mild-asthma	151/2,486 (6.1%)	521/9,944 (5.2%)	1.17 (0.97-1.41)	0.096	1.12 (0.93-1.36)	0.242	1.11 (0.91-1.35)	0.299
Severe-asthma	18/2,486 (0.7%)	60/9,944 (0.6%)	1.21 (0.72-2.06)	0.475	1.18 (0.69-2.01)	0.558	1.12 (0.65-1.93)	0.682
**COPD**								
Non-COPD	2,424/2,486 (97.5%)	9,762/9,944 (98.2%)	1		1		1	
Mild- COPD	34/2,486 (1.4%)	110/9,944 (1.1%)	1.25 (0.85-1.83)	0.267	1.26 (0.85-1.88)	0.251	1.23 (0.83-1.84)	0.303
Severe- COPD	28/2,486 (1.1%)	72/9,944 (0.7%)	1.57 (1.01-2.43)	0.045*	1.49 (0.95-2.33)	0.083	1.47 (0.94-2.30)	0.094
**Age ≥ 60 years old**	**n =1580**	**n =6320**						
**Asthma**								
Non-asthma	1,387/1,580 (87.8%)	5,604/6,320 (88.7%)	1		1		1	
Mild-asthma	171/1,580 (10.8%)	640/6,320 (10.1%)	1.08 (0.90-1.29)	0.399	1.08 (0.89-1.30)	0.437	1.06 (0.88-1.29)	0.536
Severe-asthma	22/1,580 (1.4%)	76/6,320 (1.2%)	1.17 (0.73-1.89)	0.520	1.21 (0.73-1.98)	0.461	1.17 (0.70-1.93)	0.553
**COPD**								
Non-COPD	1478/1,580 (93.5%)	5,963/6,320 (94.4%)	1		1		1	
Mild- COPD	67/1,580 (4.2%)	249/6,320 (3.9%)	1.09 (0.83-1.43)	0.554	1.07 (0.80-1.43)	0.640	1.04 (0.78-1.40)	0.784
Severe- COPD	35/1,580 (2.2%)	108/6,320 (1.7%)	1.31 (0.89-1.93)	0.171	1.20 (0.80-1.80)	0.389	1.16 (0.76-1.75)	0.497
**Men (n = 7,630)**								
**Asthma**								
Non-asthma	1,406/1,526 (92.1%)	5,722/6,104 (93.7%)	1		1		1	
Mild-asthma	106/1,526 (6.9%)	325/6,104 (5.3%)	1.34 (1.06-1.68)	0.013*	1.29 (1.01-1.64)	0.038*	1.30 (1.01-1.66)	0.039*
Severe-asthma	14/1,526 (0.9%)	57/6,104 (0.9%)	1.01 (0.56-1.81)	0.986	0.93 (0.50-1.72)	0.812	0.93 (0.50-1.74)	0.815
**COPD**								
Non-COPD	1,457/1,526 (95.5%)	5,864/6,104 (96.1%)	1		1		1	

Mild- COPD	39/1,526 (2.6%)	162/6,104 (2.7%)	0.97 (0.68-1.39)	0.878	0.93 (0.64-1.35)	0.701	0.87 (0.59-1.28)	0.482
Severe- COPD	30/1,526 (2.0%)	78/6,104 (1.3%)	1.55 (1.01-2.38)	0.044*	1.23 (0.78-1.94)	0.375	1.16 (0.73-1.85)	0.528
**Women (n = 12,700)**								
**Asthma**								
Non-asthma	2,298/2,540 (90.5%)	9,245/10,160 (91.0%)	1		1		1	
Mild-asthma	216/2,540 (8.5%)	836/10,160 (8.2%)	1.04 (0.89-1.22)	0.624	1.03 (0.88-1.21)	0.704	1.01 (0.86-1.19)	0.899
Severe-asthma	26/2,540 (1.0%)	79/10,160 (0.8%)	1.33 (0.85-2.07)	0.216	1.41 (0.89-2.22)	0.140	1.33 (0.84-2.10)	0.229
**COPD**								
Non-COPD	2,445/2,540 (96.3%)	9,861/10,160 (97.1%)	1		1		1	
Mild- COPD	62/2,540 (2.4%)	197/10,160 (1.9%)	1.27 (0.95-1.70)	0.103	1.29 (0.96-1.74)	0.093	1.27 (0.94-1.71)	0.127
Severe- COPD	33/2,540 (1.3%)	102/10,160 (1.0%)	1.31 (0.88-1.94)	0.184	1.39 (0.93-2.08)	0.111	1.36 (0.91-2.05)	0.134
**Low income (n = 7,360)**								
**Asthma**								
Non-asthma	1,343/1,472 (91.2%)	5,396/5,888 (91.6%)	1		1		1	
Mild-asthma	116/1,472 (7.9%)	436/5,888 (7.4%)	1.07 (0.86-1.33)	0.538	1.05 (0.84-1.32)	0.662	1.03 (0.82-1.29)	0.800
Severe-asthma	13/1,472 (0.9%)	56/5,888 (1.0%)	0.93 (0.51-1.71)	0.823	1.01 (0.54-1.88)	0.981	0.95 (0.51-1.79)	0.877
**COPD**								
Non-COPD	1,415/1,472 (96.1%)	5,690/5,888 (96.6%)	1		1		1	
Mild- COPD	36/1,472 (2.4%)	134/5,888 (2.3%)	1.08 (0.75-1.58)	0.677	1.20 (0.81-1.76)	0.362	1.19 (0.80-1.77)	0.381
Severe- COPD	21/1,472 (1.4%)	64/5,888 (1.1%)	1.32 (0.80-2.17)	0.272	1.28 (0.76-2.16)	0.349	1.28 (0.76-2.16)	0.353
**Middle income (n = 6,245)**								
**Asthma**								
Non-asthma	1,142/1,249 (91.4%)	4,652/4,996 (93.1%)	1		1		1	
Mild-asthma	94/1,249 (7.5%)	315/4,996 (6.3%)	1.22 (0.96-1.55)	0.106	1.21 (0.95-1.55)	0.130	1.21 (0.94-1.55)	0.137
Severe-asthma	13/1,249 (1.0%)	29/4,996 (0.6%)	1.83 (0.95-3.53)	0.071	1.52 (0.77-3.02)	0.230	1.55 (0.77-3.10)	0.216
**COPD**								
Non-COPD	1,206/1,249 (96.6%)	4,843/4,996 (96.9%)	1		1		1	
Mild- COPD	26/1,249 (2.1%)	101/4,996 (2.0%)	1.04 (0.67-1.61)	0.876	0.96 (0.61-1.52)	0.863	0.90 (0.56-1.44)	0.657
Severe- COPD	17/1,249 (1.4%)	52/4,996 (1.0%)	1.31 (0.76-2.28)	0.332	1.26 (0.72-2.22)	0.424	1.20 (0.68-2.13)	0.524
**High income (n = 6,725)**								

**Asthma**								
Non-asthma	1,219/1,345 (90.6%)	4,919/5,380 (91.4%)	1		1		1	
Mild-asthma	112/1,345 (8.3%)	410/5,380 (7.6%)	1.11 (0.89-1.38)	0.376	1.07 (0.85-1.34)	0.561	1.04 (0.82-1.31)	0.771
Severe-asthma	14/1,345 (1.0%)	51/5,380 (0.9%)	1.11 (0.61-2.01)	0.733	1.17 (0.64-2.14)	0.617	1.06 (0.57-1.98)	0.851
**COPD**								
Non-COPD	1,281/1,345 (95.2%)	5,192/5,380 (96.5%)	1		1		1	
Mild- COPD	39/1,345 (2.9%)	124/5,380 (2.3%)	1.28 (0.89-1.85)	0.184	1.19 (0.82-1.74)	0.365	1.18 (0.80-1.73)	0.402
Severe- COPD	25/1,345 (1.9%)	64/5,380 (1.2%)	1.59 (1.00-2.54)	0.051	1.44 (0.88-2.35)	0.146	1.41 (0.85-2.34)	0.179
**Underweight (n = 700)**								
**Asthma**								
Non-asthma	119/129 (92.2%)	532/571 (93.2%)	1		1		1	
Mild-asthma	8/129 (6.2%)	36/571 (6.3%)	0.99 (0.45-2.19)	0.987	1.03 (0.44-2.43)	0.939	0.56 (0.20-1.55)	0.265
Severe-asthma	2/129 (1.6%)	3/571 (0.5%)	2.98 (0.49-18.03)	0.234	3.27 (0.49-21.72)	0.220	3.65 (0.54-24.58)	0.184
**COPD**								
Non-COPD	119/129 (92.2%)	558/571 (97.7%)	1		1		1	
Mild- COPD	8/129 (6.2%)	8/571 (1.4%)	4.69 (1.73-12.74)	0.003*	9.58 (2.96-31.03)	0.001*	12.56 (3.62-43.59)	<0.001*
Severe- COPD	2/129 (1.6%)	5/571 (0.9%)	1.88 (0.36-9.78)	0.456	2.63 (0.36-19.03)	0.338	2.72 (0.39-19.01)	0.314
**Normal weight (n =7,819)**								
**Asthma**								
Non-asthma	1,339/1,458 (91.8%)	5,901/6,361 (92.8%)	1		1		1	
Mild-asthma	106/1,458 (7.3%)	413/6,361 (6.5%)	1.13 (0.91-1.41)	0.276	1.17 (0.93-1.47)	0.181	1.13 (0.89-1.42)	0.312
Severe-asthma	13/1,458 (0.9%)	47/6,361 (0.7%)	1.22 (0.66-2.26)	0.529	1.33 (0.70-2.53)	0.383	1.22 (0.63-2.34)	0.557
**COPD**								
Non-COPD	1,397/1,458 (95.8%)	6,171/6,361 (97.0%)	1		1		1	
Mild- COPD	34/1,458 (2.3%)	127/6,361 (2.0%)	1.18 (0.81-1.73)	0.391	1.16 (0.77-1.73)	0.477	1.11 (0.74-1.67)	0.617
Severe- COPD	27/1,458 (1.9%)	63/6,361 (1.0%)	1.89 (1.20-2.98)	0.006*	1.95 (1.22-3.13)	0.006*	1.88 (1.17-3.03)	0.010*
**Overweight (n = 4,868)**								
**Asthma**								
Non-asthma	923/1,010 (91.4%)	3,569/3,858 (92.5%)	1		1		1	
Mild-asthma	77/1,010 (7.6%)	263/3,858 (6.8%)	1.13 (0.87-1.47)	0.357	1.11 (0.85-1.47)	0.442	1.11 (0.84-1.47)	0.458

Severe-asthma	10/1,010 (1.0%)	26/3,858 (0.7%)	1.49 (0.72-3.10)	0.288	1.25 (0.59-2.68)	0.564	1.24 (0.57-2.71)	0.583
**COPD**								
Non-COPD	970/1,010 (96.0%)	3,725/3,858 (96.6%)	1		1		1	
Mild- COPD	27/1,010 (2.7%)	94/3,858 (2.4%)	1.10 (0.72-1.70)	0.658	1.08 (0.69-1.71)	0.731	1.05 (0.66-1.66)	0.844
Severe- COPD	13/1,010 (1.3%)	39/3,858 (1.0%)	1.28 (0.68-2.41)	0.443	1.05 (0.54-2.02)	0.896	0.99 (0.50-1.94)	0.964
**Obese (n = 6,943)**								
**Asthma**								
Non-asthma	1,323/1,469 (90.1%)	4,965/5,474 (90.7%)	1		1		1	
Mild-asthma	131/1,469 (8.9%)	449/5,474 (8.2%)	1.10 (0.89-1.34)	0.383	1.03 (0.83-1.27)	0.804	1.04 (0.83-1.29)	0.747
Severe-asthma	15/1,469 (1.0%)	60/5,474 (1.1%)	0.94 (0.53-1.66)	0.826	0.96 (0.54-1.71)	0.886	0.98 (0.55-1.76)	0.940
**COPD**								
Non-COPD	1,416/1,469 (96.4%)	5,271/5,474 (96.3%)	1		1		1	
Mild- COPD	32/1,469 (2.2%)	130/5,474 (2.4%)	0.92 (0.62-1.36)	0.661	0.91 (0.61-1.36)	0.635	0.90 (0.60-1.36)	0.617
Severe- COPD	21/1,469 (1.4%)	73/5,474 (1.3%)	1.07 (0.66-1.75)	0.784	0.96 (0.58-1.60)	0.881	0.96 (0.57-1.60)	0.869
**Non-smoker (n = 14,753)**								
**Asthma**								
Non-asthma	2,906/3,181 (91.4%)	10,609/11,572 (91.7%)	1		1		1	
Mild-asthma	247/3,181 (7.8%)	880/11,572 (7.6%)	1.03 (0.89-1.19)	0.745	1.04 (0.89-1.21)	0.604	1.02 (0.88-1.19)	0.796
Severe-asthma	28/3,181 (0.9%)	83/11,572 (0.7%)	1.23 (0.80-1.89)	0.343	1.28 (0.82-2.00)	0.270	1.22 (0.78-1.90)	0.393
**COPD**								
Non-COPD	3,062/3,181 (96.3%)	11,225/11,572 (97.0%)	1		1		1	
Mild- COPD	72/3,181 (2.3%)	223/11,572 (1.9%)	1.18 (0.91-1.55)	0.219	1.17 (0.89-1.55)	0.263	1.16 (0.87-1.53)	0.316
Severe- COPD	47/3,181 (1.5%)	124/11,572 (1.1%)	1.39 (0.99-1.95)	0.056	1.39 (0.98-1.97)	0.063	1.37 (0.96-1.94)	0.080
**Past or current smoker (n = 5,577)**								
**Asthma**								
Non-asthma	798/885 (90.2%)	4,358/4,692 (92.9%)	1		1		1	
Mild-asthma	75/885 (8.5%)	281/4,692 (6.0%)	1.46 (1.12-1.90)	0.005*	1.36 (1.03-1.79)	0.032*	1.37 (1.03-1.83)	0.031*
Severe-asthma	12/885 (1.4%)	53/4,692 (1.1%)	1.24 (0.66-2.32)	0.510	1.21 (0.63-2.32)	0.565	1.23 (0.63-2.41)	0.537
**COPD**								

Non-COPD	840/885 (94.9%)	4,500/4,692 (95.9%)	1		1		1	
Mild-COPD	29/885 (3.3%)	136/4,692 (2.9%)	1.14 (0.76-1.72)	0.522	1.00 (0.65-1.53)	0.999	0.90 (0.58-1.39)	0.623
Severe-COPD	16/885 (1.8%)	56/4,692 (1.2%)	1.53 (0.88-2.68)	0.136	1.17 (0.64-2.11)	0.616	1.04 (0.56-1.92)	0.907
**Alcohol consumption < 1 time a week (n = 13,465)**								
**Asthma**								
Non-asthma	2,676/2,968 (90.2%)	9,534/10,497 (90.8%)	1		1		1	
Mild-asthma	259/2,968 (8.7%)	855/10,497 (8.1%)	1.08 (0.93-1.25)	0.304	1.10 (0.95-1.28)	0.217	1.09 (0.93-1.27)	0.298
Severe-asthma	33/2,968 (1.1%)	108/10,497 (1.0%)	1.09 (0.74-1.61)	0.671	1.17 (0.78-1.75)	0.441	1.13 (0.75-1.70)	0.563
**COPD**								
Non-COPD	2,842/2,968 (95.8%)	10,107/10,497 (96.3%)	1		1		1	
Mild- COPD	78/2,968 (2.6%)	263/10,497 (2.5%)	1.06 (0.82-1.36)	0.684	1.10 (0.84-1.43)	0.500	1.07 (0.81-1.40)	0.637
Severe-COPD	48/2,968 (1.6%)	127/10,497 (1.2%)	1.34 (0.96-1.88)	0.083	1.30 (0.92-1.85)	0.137	1.27 (0.89-1.80)	0.187
**Alcohol consumption ≥1 time a week (n = 6,865)**								
**Asthma**								
Non-asthma	1,028/1,098 (93.6%)	5,433/5,767 (94.2%)	1		1		1	
Mild-asthma	63/1,098 (5.7%)	306/5,767 (5.3%)	1.09 (0.82-1.44)	0.554	1.09 (0.82-1.45)	0.562	1.06 (0.79-1.42)	0.695
Severe-asthma	7/1,098 (0.6%)	28/5,767 (0.5%)	1.32 (0.58-3.03)	0.511	1.31 (0.55-3.13)	0.547	1.20 (0.50-2.91)	0.682
**COPD**								
Non-COPD	1,060/1,098 (96.5%)	5,618/5,767 (97.4%)	1		1		1	
Mild- COPD	23/1,098 (2.1%)	96/5,767 (1.7%)	1.27 (0.80-2.01)	0.308	1.21 (0.75-1.95)	0.444	1.18 (0.72-1.92)	0.514
Severe-COPD	15/1,098 (1.4%)	53/5,767 (0.9%)	1.50 (0.84-2.67)	0.167	1.42 (0.78-2.59)	0.256	1.38 (0.75-2.54)	0.297
**Systolic blood pressure < 140 mmHg and diastolic blood pressure < 90 mmHg (n = 17,427)**								
**Asthma**								
Non-asthma	3,218/3,530 (91.2%)	12,834/13,897 (92.4%)	1		1		1	
Mild-asthma	280/3,530 (7.9%)	948/13,897 (6.8%)	1.18 (1.03-1.35)	0.021*	1.15 (1.00-1.33)	0.056	1.13 (0.98-1.31)	0.099
Severe-asthma	32/3,530 (0.9%)	115/13,897 (0.8%)	1.11 (0.75-1.65)	0.604	1.10 (0.74-1.66)	0.632	1.05 (0.70-1.58)	0.817
**COPD**								
Non-COPD	3,395/3,530 (96.2%)	13,466/13,897 (96.9%)	1		1		1	
Mild-COPD	81/3,530 (2.3%)	279/13,897 (2.0%)	1.15 (0.90-1.48)	0.269	1.13 (0.87-1.46)	0.365	1.10 (0.84-1.43)	0.500
Severe-COPD	54/3,530 (1.5%)	152/13,897 (1.1%)	1.41 (1.03-1.93)	0.032*	1.37 (0.99-1.90)	0.058	1.33 (0.96-1.85)	0.092

**Systolic blood pressure ≥ 140 mmHg or diastolic blood pressure ≥90 mmHg (n = 2,903)**								
**Asthma**								
Non-asthma	486/536 (90.7%)	2,133/2,367 (90.1%)	1		1		1	
Mild-asthma	42/536 (7.8%)	213/2,367 (9.0%)	0.87 (0.61-1.22)	0.412	0.79 (0.55-1.15)	0.216	0.79 (0.54-1.14)	0.203
Severe-asthma	8/536 (1.5%)	21/2,367 (0.9%)	1.68 (0.74-3.80)	0.218	1.73 (0.74-4.04)	0.209	1.68 (0.70-4.00)	0.245
**COPD**								
Non-COPD	507/536 (94.6%)	2,259/2,367 (95.4%)	1		1		1	
Mild-COPD	20/536 (3.7%)	80/2,367 (3.4%)	1.11 (0.68-1.84)	0.672	1.07 (0.63-1.82)	0.791	1.08 (0.62-1.86)	0.792
Severe-COPD	9/536 (1.7%)	28/2,367 (1.2%)	1.43 (0.67-3.06)	0.352	1.07 (0.48-2.40)	0.866	1.08 (0.48-2.43)	0.851
**Fasting blood glucose < 100 mg/dL (n = 12,806)**								
**Asthma**								
Non-asthma	2,309/2,522 (91.6%)	9,465/10,284 (92.0%)	1		1		1	
Mild-asthma	192/2,522 (7.6%)	730/10,284 (7.1%)	1.08 (0.91-1.27)	0.373	1.04 (0.88-1.24)	0.635	1.02 (0.85-1.21)	0.870
Severe-asthma	21/2,522 (0.8%)	89/10,284 (0.9%)	0.97 (0.60-1.56)	0.891	0.93 (0.57-1.52)	0.773	0.87 (0.53-1.42)	0.578
**COPD**								
Non-COPD	2,424/2,522 (96.1%)	9,967/10,284 (96.9%)	1		1		1	
Mild-COPD	58/2,522 (2.3%)	201/10,284 (2.0%)	1.19 (0.88-1.59)	0.257	1.20 (0.88-1.62)	0.250	1.20 (0.88-1.64)	0.243
Severe-COPD	40/2,522 (1.6%)	116/10,284 (1.1%)	1.42 (0.99-2.04)	0.059	1.40 (0.96-2.04)	0.078	1.41 (0.97-2.07)	0.075
**Fasting blood glucose ≥ 100 mg/dL (n =7,524)**								
**Asthma**								
Non-asthma	1,395/1,544 (90.3%)	5,502/5,980 (92.0%)	1		1		1	
Mild-asthma	130/1,544 (8.4%)	431/5,980 (7.2%)	1.19 (0.97-1.46)	0.096	1.19 (0.96-1.48)	0.107	1.20 (0.96-1.49)	0.106
Severe-asthma	19/1,544 (1.2%)	47/5,980 (0.8%)	1.60 (0.93-2.73)	0.088	1.67 (0.95-2.93)	0.077	1.68 (0.94-2.99)	0.078
**COPD**								
Non-COPD	1,478/1,544 (95.7%)	5,758/5,980 (96.3%)	1		1		1	
Mild-COPD	43/1,544 (2.8%)	158/5,980 (2.6%)	1.06 (0.75-1.49)	0.737	1.03 (0.72-1.48)	0.866	0.94 (0.65-1.36)	0.733
Severe-COPD	23/1,544 (1.5%)	64/5,980 (1.1%)	1.40 (0.87-2.26)	0.169	1.17 (0.71-1.94)	0.541	1.09 (0.65-1.82)	0.741
**Total cholesterol < 200 mg/dL (n = 11,393)**								
**Asthma**								
Non-asthma	2,109/2,325 (90.7%)	8,324/9,068 (91.8%)	1		1		1	

Mild-asthma	190/2,325 (8.2%)	661/9,068 (7.3%)	1.14 (0.96-1.34)	0.142	1.14 (0.96-1.36)	0.145	1.12 (0.93-1.34)	0.227
Severe-asthma	26/2,325 (1.1%)	83/9,068 (0.9%)	1.24 (0.79-1.93)	0.348	1.26 (0.80-1.99)	0.327	1.19 (0.75-1.89)	0.469
**COPD**								
Non-COPD	2,224/2,325 (95.7%)	8,744/9,068 (96.4%)	1		1		1	
Mild-COPD	63/2,325 (2.7%)	228/9,068 (2.5%)	1.09 (0.82-1.44)	0.566	1.12 (0.83-1.51)	0.458	1.02 (0.83-1.26)	0.841
Severe-COPD	38/2,325 (1.6%)	96/9,068 (1.1%)	1.56 (1.07-2.27)	0.022*	1.46 (0.98-2.17)	0.061	1.03 (0.55-1.90)	0.938
**Total cholesterol ≥ 200 mg/dL (n = 8,937)**								
**Asthma**								
Non-asthma	1,595/1,741 (91.6%)	6,643/7,196 (92.3%)	1		1		1	
Mild-asthma	132/1,741 (7.6%)	500/7,196 (6.9%)	1.10 (0.90-1.34)	0.351	1.04 (0.84-1.28)	0.733	1.08 (0.80-1.46)	0.622
Severe-asthma	14/1,741 (0.8%)	53/7,196 (0.7%)	1.10 (0.61-1.99)	0.752	1.07 (0.58-1.96)	0.830	1.40 (0.94-2.09)	0.101
**COPD**								
Non-COPD	1,678/1,741 (96.4%)	6,981/7,196 (97.0%)	1		1		1	
Mild-COPD	38/1,741 (2.2%)	131/7,196 (1.8%)	1.21 (0.84-1.74)	0.313	1.17 (0.80-1.71)	0.410	1.17 (0.79-1.71)	0.436
Severe-COPD	25/1,741 (1.4%)	84/7,196 (1.2%)	1.24 (0.79-1.94)	0.352	1.16 (0.73-1.85)	0.537	1.15 (0.72-1.85)	0.554
**CCI scores = 0 (n = 17,698)**								
**Asthma**								
Non-asthma	2,840/3,089 (91.9%)	13,489/14,609 (92.3%)	1		1		1	
Mild-asthma	219/3,089 (7.1%)	1004/14,609 (6.9%)	1.04 (0.89-1.21)	0.647	1.05 (0.90-1.23)	0.543	1.04 (0.89-1.22)	0.634
Severe-asthma	30/3,089 (1.0%)	116/14,609 (0.8%)	1.23 (0.82-1.84)	0.318	1.20 (0.79-1.81)	0.387	1.16 (0.76-1.75)	0.497
**COPD**								
Non-COPD	2,990/3,089 (96.8%)	14,172/14,609 (97.0%)	1		1		1	
Mild-COPD	58/3,089 (1.9%)	298/14,609 (2.0%)	0.92 (0.69-1.23)	0.578	1.03 (0.77-1.37)	0.855	1.01 (0.76-1.36)	0.939
Severe-COPD	41/3,089 (1.3%)	139/14,609 (1.0%)	1.40 (0.99-1.99)	0.061	1.37 (0.96-1.97)	0.083	1.35 (0.94-1.93)	0.108
**CCI scores = 1 (n = 1,454)**								
**Asthma**								
Non-asthma	524/588 (89.1%)	782/866 (90.3%)	1		1		1	
Mild-asthma	57/588 (9.7%)	74/866 (8.5%)	1.15 (0.80-1.65)	0.451	1.37 (0.93-2.01)	0.401	1.28 (0.86-1.90)	0.398
Severe-asthma	7/588 (1.2%)	10/866 (1.2%)	1.05 (0.40-2.76)	0.930	1.32 (0.48-3.69)	0.109	1.14 (0.40-3.29)	0.226

**COPD**								
Non-COPD	555/588 (94.4%)	824/866 (95.2%)	1		1		1	
Mild-COPD	22/588 (3.7%)	24/866 (2.8%)	1.36 (0.76-2.45)	0.305	1.69 (0.91-3.12)	0.399	1.52 (0.80-2.89)	0.803
Severe-COPD	11/588 (1.9%)	18/866 (2.1%)	0.91 (0.43-1.94)	0.801	1.21 (0.54-2.69)	0.095	1.14 (0.51-2.57)	0.204
**CCI scores ≥ 2 (n = 1,178)**								
**Asthma**								
Non-asthma	340/389 (87.4%)	696/789 (88.2%)	1		1		1	
Mild-asthma	46/389 (11.8%)	83/789 (10.5%)	1.14 (0.77-1.66)	0.518	1.16 (0.77-1.73)	0.114	1.13 (0.74-1.72)	0.114
Severe-asthma	3/389 (0.8%)	10/789 (1.3%)	0.61 (0.17-2.25)	0.462	0.55 (0.15-2.09)	0.424	0.52 (0.13-2.02)	0.411
**COPD**								
Non-COPD	357/389 (91.8%)	729/789 (92.4%)	1		1		1	
Mild-COPD	21/389 (5.4%)	37/789 (4.7%)	1.16 (0.67-2.01)	0.599	1.12 (0.63-1.98)	0.146	1.15 (0.64-2.08)	0.566
Severe-COPD	11/389 (2.8%)	23/789 (2.9%)	0.98 (0.47-2.03)	0.949	1.07 (0.50-2.30)	0.416	1.05 (0.48-2.32)	0.344
**NSAID used < 6 times (n = 10,717)**								
**Asthma**								
Non-asthma	1,871/1,972 (94.9%)	8,318/8,745 (95.1%)	1		1		1	
Mild-asthma	96/1,972 (4.9%)	376/8,745 (4.3%)	1.14 (0.90-1.43)	0.280	1.16 (0.91-1.47)	0.232	1.12 (0.88-1.43)	0.364
Severe-asthma	05/1,972 (0.3%)	51/8,745 (0.6%)	0.44 (0.17-1.09)	0.077	0.51 (0.20-1.30)	0.161	0.48 (0.19-1.24)	0.128
**COPD**								
Non-COPD	1,912/1,972 (97.0%)	8,547/8,745 (97.7%)	1		1		1	
Mild-COPD	35/1,972 (1.8%)	136/8,745 (1.6%)	1.15 (0.79-1.67)	0.464	1.10 (0.74-1.63)	0.641	1.04 (0.89-1.23)	0.611
Severe-COPD	25/1,972 (1.3%)	62/8,745 (0.7%)	1.80 (1.13-2.88)	0.013*	1.66 (1.02-2.71)	0.040*	1.43 (0.94-2.18)	0.092
**NSAID used ≥ 6 times (n = 9,613)**								
**Asthma**								
Non-asthma	1,833/2,094 (87.5%)	6,649/7,519 (88.4%)	1		1		1	
Mild-asthma	226/2,094 (10.8%)	785/7,519 (10.4%)	1.04 (0.89-1.22)	0.588	1.05 (0.89-1.24)	0.543	1.10 (0.73-1.64)	0.655
Severe-asthma	35/2,094 (1.7%)	85/7,519 (1.1%)	1.49 (1.01-2.22)	0.047*	1.47 (0.98-2.21)	0.066	1.66 (1.02-2.72)	0.042*
**COPD**								
Non-COPD	1,990/2,094 (95.0%)	7,178/7,519 (95.5%)	1		1		1	
Mild-COPD	66/2,094 (3.2%)	223/7,519 (3.0%)	1.07 (0.81-1.41)	0.646	1.12 (0.84-1.49)	0.455	1.07 (0.80-1.44)	0.644

Severe-COPD	38/2,094 (1.8%)	118/7,519 (1.6%)	1.16 (0.80-1.68)	0.426	1.14 (0.78-1.68)	0.496	1.09 (0.74-1.61)	0.674
**Steroid used < 3 times (n = 11,308)**								
**Asthma**								
Non-asthma	2,101/2,206 (95.2%)	8,676/9,102 (95.3%)	1		1		1	
Mild-asthma	98/2,206 (4.4%)	405/9,102 (4.4%)	1.00 (0.80-1.25)	0.995	0.97 (0.77-1.23)	0.816	0.98 (0.78-1.24)	0.879
Severe-asthma	7/2,206 (0.3%)	21/9,102 (0.2%)	1.38 (0.59-3.24)	0.464	1.41 (0.59-3.37)	0.439	1.45 (0.60-3.46)	0.409
**COPD**								
Non-COPD	2,164/2,206 (98.1%)	8,927/9,102 (98.1%)	1		1		1	
Mild-COPD	31/2,206 (1.4%)	143/9,102 (1.6%)	0.89 (0.61-1.32)	0.576	0.85 (0.57-1.28)	0.445	0.85 (0.56-1.28)	0.434
Severe-COPD	11/2,206 (0.5%)	32/9,102 (0.4%)	1.42 (0.71-2.82)	0.318	1.26 (0.62-2.55)	0.522	1.26 (0.62-2.56)	0.515
**Steroid used ≥ 3 times (n = 9,022)**								
**Asthma**								
Non-asthma	1,603/1,860 (86.2%)	6,291/7,162 (87.8%)	1		1		1	
Mild-asthma	224/1,860 (12.0%)	756/7,162 (10.6%)	1.16 (0.99-1.36)	0.063	1.14 (0.96-1.34)	0.131	1.10 (0.93-1.30)	0.253
Severe-asthma	33/1,860 (1.8%)	115/7,162 (1.6%)	1.13 (0.76-1.67)	0.551	1.12 (0.75-1.68)	0.570	1.04 (0.69-1.57)	0.858
**COPD**								
Non-COPD	1,738/1,860 (93.4%)	6,798/7,162 (94.9%)	1		1		1	
Mild-COPD	70/1,860 (3.8%)	216/7,162 (3.0%)	1.27 (0.96-1.67)	0.091	1.28 (0.96-1.70)	0.096	1.25 (0.93-1.67)	0.140
Severe-COPD	52/1,860 (2.8%)	148/7,162 (2.1%)	1.38 (1.00-1.89)	0.052	1.34 (0.96-1.87)	0.086	1.31 (0.93-1.83)	0.122
**Non-hypertension (n = 14,543)**								
**Asthma**								
Non-asthma	2,662/2,878 (92.5%)	10,868/11,665 (93.2%)	1		1		1	
Mild-asthma	194/2,878 (6.7%)	711/11,665 (6.1%)	1.11 (0.95-1.31)	0.198	1.12 (0.94-1.32)	0.200	1.09 (0.92-1.30)	0.313
Severe-asthma	22/2,878 (0.8%)	86/11,665 (0.7%)	1.04 (0.65-1.67)	0.856	1.11 (0.69-1.81)	0.660	1.05 (0.65-1.71)	0.832
**COPD**								
Non-COPD	2,784/2,878 (96.7%)	11,367/11,665 (97.4%)	1		1		1	
Mild-COPD	55/2,878 (1.9%)	187/11,665 (1.6%)	1.20 (0.89-1.63)	0.237	1.21 (0.88-1.65)	0.245	1.18 (0.86-1.63)	0.297
Severe-COPD	39/2,878 (1.4%)	111/11,665 (1.0%)	1.44 (0.99-2.07)	0.054	1.48 (1.01-2.16)	0.043*	1.45 (0.99-2.12)	0.058
**Hypertension (n = 5,787)**								
**Asthma**								

Non-asthma	1,042/1,188 (87.7%)	4,099/4,599 (89.1%)	1		1		1	
Mild-asthma	128/1,188 (10.8%)	450/4,599 (9.8%)	1.12 (0.91-1.38)	0.289	1.08 (0.86-1.34)	0.516	1.08 (0.86-1.35)	0.520
Severe-asthma	18/1,188 (1.5%)	50/4,599 (1.1%)	1.42 (0.82-2.44)	0.209	1.33 (0.76-2.34)	0.321	1.33 (0.75-2.39)	0.333
**COPD**								
Non-COPD	1,118/1,188 (94.1%)	4,358/4,599 (94.8%)	1		1		1	
Mild-COPD	46/1,188 (3.9%)	172/4,599 (3.7%)	1.04 (0.75-1.45)	0.806	1.02 (0.72-1.45)	0.898	0.97 (0.68-1.39)	0.876
Severe-COPD	24/1,188 (2.0%)	69/4,599 (1.5%)	1.36 (0.85-2.17)	0.203	1.11 (0.67-1.82)	0.691	1.06 (0.64-1.75)	0.832

* Conditional or unconditional logistic regression model, Significance at $P < 0.05$

† Stratified model for age, sex and income.

‡ Model 1 was adjusted for obesity, smoking, alcohol consumption, systolic blood pressure, diastolic blood pressure, fasting blood glucose, total cholesterol, CCI scores, number of NSAID used, number of steroid used, and hypertension.

§ Model 2 was adjusted for model 1 plus asthma and COPD.

Table S2. Subgroup analyses of crude and adjusted odds ratios of asthma and COPD for morbidity in COVID-19 participants by covariates

Characteristics	Severe participants (exposure/total, %)	Mild participants (exposure/total, %)	ORs (95% confidence interval) for morbidity					
			Crude	P-value	Model 1†	P-value	Model 2‡	P-value
**Age < 60 years old (n = 2,486)**								
**Asthma**								
Non-asthma	82/92 (89.1%)	2,235/2,394 (93.4%)	1		1		1	
Mild-asthma	10/92 (10.9%)	141/2,394 (5.9%)	1.93 (0.98-3.81)	0.057	2.05 (1.01-4.18)	0.047*	2.18 (1.06-4.50)	0.035*
Severe-asthma	0/92 (0.0%)	18/2,394 (0.8%)	N/A		N/A		N/A	
**COPD**								
Non-COPD	89/92 (96.7%)	2,335/2,394 (97.5%)	1		1		1	
Mild-COPD	1/92 (1.1%)	33/2,394 (1.4%)	0.80 (0.11-5.88)	0.822	0.76 (0.10-5.74)	0.788	0.53 (0.07-4.26)	0.552
Severe-COPD	2/92 (2.2%)	26/2,394 (1.1%)	2.02 (0.47-8.64)	0.344	1.98 (0.44-8.99)	0.376	2.09 (0.46-9.53)	0.341
**Age ≥ 60 years old (n = 1,580)**								
**Asthma**								
Non-asthma	214/251 (85.3%)	1,173/1,329 (88.3%)	1		1		1	
Mild-asthma	33/251 (13.1%)	138/1,329 (10.4%)	1.31 (0.87-1.97)	0.192	0.92 (0.58-1.47)	0.723	0.83 (0.51-1.35)	0.445
Severe-asthma	4/251 (1.6%)	18/1,329 (1.4%)	1.22 (0.41-3.63)	0.724	0.61 (0.16-2.29)	0.468	0.46 (0.11-1.87)	0.278
**COPD**								
Non-COPD	224/251 (89.2%)	1,254/1,329 (94.4%)	1		1		1	
Mild-COPD	14/251 (5.6%)	53/1,329 (4.0%)	1.48 (0.81-2.71)	0.206	1.01 (0.50-2.04)	0.982	1.16 (0.56-2.41)	0.689
Severe-COPD	13/251 (5.2%)	22/1,329 (1.7%)	3.31 (1.64-6.66)	0.001*	2.35 (1.02-5.41)	0.045*	2.70 (1.14-6.42)	0.024*
**Men (n = 1,526)**								
**Asthma**								
Non-asthma	181/204 (88.7%)	1,225/1,322 (92.7%)	1		1		1	
Mild-asthma	21/204 (10.3%)	85/1,322 (6.4%)	1.67 (1.01-2.76)	0.045*	0.83 (0.46-1.49)	0.531	0.70 (0.37-1.30)	0.253

Severe-asthma	2/204 (1.0%)	12/1,322 (0.9%)	1.13 (0.25-5.08)	0.875	0.31 (0.04-2.34)	0.259	0.18 (0.02-1.53)	0.116
**COPD**								
Non-COPD	183/204 (89.7%)	1,274/1,322 (96.4%)	1		1		1	
Mild-COPD	12/204 (5.9%)	27/1,322 (2.0%)	3.09 (1.54-6.22)	0.002*	1.78 (0.76-4.15)	0.181	2.30 (0.95-5.58)	0.066
Severe-COPD	9/204 (4.4%)	21/1,322 (1.6%)	2.98 (1.35-6.62)	0.007*	1.63 (0.62-4.26)	0.322	2.03 (0.76-5.44)	0.159
**Women (n = 2,540)**								
**Asthma**								
Non-asthma	115/139 (82.7%)	2,183/2,401 (90.9%)	1		1		1	
Mild-asthma	22/139 (15.8%)	194/2,401 (8.1%)	2.15 (1.33-3.48)	0.002*	1.49 (0.87-2.54)	0.149	0.00 (0.00-0.00)	0.000
Severe-asthma	2/139 (1.4%)	24/2,401 (1.0%)	1.58 (0.37-6.78)	0.537	0.93 (0.19-4.57)	0.923	1.46 (0.84-2.56)	0.184
**COPD**								
Non-COPD	130/139 (93.5%)	2,315/2,401 (96.4%)	1		1		1	
Mild-COPD	3/139 (2.2%)	59/2,401 (2.5%)	0.91 (0.28-2.93)	0.868	0.42 (0.12-1.46)	0.171	0.37 (0.10-1.35)	0.133
Severe-COPD	6/139 (4.3%)	27/2,401 (1.1%)	3.96 (1.61-9.75)	0.003*	3.11 (1.10-8.78)	0.032*	2.71 (0.93-7.96)	0.069
**Low income (n = 1,472)**								
**Asthma**								
Non-asthma	76/88 (86.4%)	1,267/1,384 (91.5%)	1		1		1	
Mild-asthma	11/88 (12.5%)	105/1,384 (7.6%)	1.75 (0.90-3.39)	0.099	1.10 (0.51-2.39)	0.810	1.28 (0.58-2.83)	0.550
Severe-asthma	1/88 (0.0%)	12/1,384 (0.0%)	1.39 (0.18-10.83)	0.754	1.98 (0.23-17.14)	0.535	2.75 (0.27-28.34)	0.396
**COPD**								
Non-COPD	82/88 (93.2%)	1,333/1,384 (96.3%)	1		1		1	
Mild-COPD	1/88 (1.1%)	35/1,384 (2.5%)	0.46 (0.06-3.43)	0.452	0.16 (0.02-1.47)	0.106	0.13 (0.01-1.25)	0.078
Severe-COPD	5/88 (5.7%)	16/1,384 (1.2%)	5.08 (1.82-14.21)	0.002*	2.51 (0.69-9.10)	0.162	2.16 (0.57-8.09)	0.255
**Middle income (n = 1,249)**								
**Asthma**								
Non-asthma	93/110 (84.5%)	1,049/1,139 (92.1%)	1		1		1	
Mild-asthma	15/110 (13.6%)	79/1,139 (6.9%)	2.14 (1.19-3.87)	0.012*	1.56 (0.77-3.13)	0.215	1.47 (0.72-3.00)	0.291

Severe-asthma	2/110 (1.8%)	11/1,139 (1.0%)	2.05 (0.45-9.39)	0.355	0.30 (0.04-2.42)	0.255	0.18 (0.02-1.99)	0.161
**COPD**								
Non-COPD	101/110 (91.8%)	1,105/1,139 (97.0%)	1		1		1	
Mild-COPD	6/110 (5.5%)	20/1,139 (1.8%)	3.28 (1.29-8.36)	0.013*	1.75 (0.50-6.14)	0.381	2.16 (0.62-7.49)	0.224
Severe-COPD	3/110 (2.7%)	14/1,139 (1.2%)	2.34 (0.66-8.29)	0.186	1.07 (0.20-5.73)	0.941	1.10 (0.20-6.16)	0.916
**High income (n = 1,345)**								
**Asthma**								
Non-asthma	127/145 (87.6%)	1,092/1,200 (91.0%)	1		1		1	
Mild-asthma	17/145 (11.7%)	95/1,200 (7.9%)	1.54 (0.89-2.66)	0.123	1.02 (0.54-1.92)	0.961	0.90 (0.47-1.76)	0.766
Severe-asthma	1/145 (0.7%)	13/1,200 (1.1%)	0.66 (0.09-5.10)	0.692	0.49 (0.05-4.40)	0.523	0.32 (0.03-3.47)	0.348
**COPD**								
Non-COPD	130/145 (89.7%)	1,151/1,200 (95.9%)	1		1		1	
Mild-COPD	8/145 (5.5%)	31/1,200 (2.6%)	2.29 (1.03-5.08)	0.042*	1.28 (0.51-3.22)	0.594	1.37 (0.53-3.52)	0.518
Severe-COPD	7/145 (4.8%)	18/1,200 (1.5%)	3.45 (1.41-8.40)	0.007*	2.88 (0.96-8.63)	0.058	3.23 (1.06-9.88)	0.040*
**Underweight (n = 129)**								
**Asthma**								
Non-asthma	5/5 (100.0%)	114/124 (91.9%)	1		1		1	
Mild-asthma	0/5 (0.0%)	8/124 (6.5%)	N/A		N/A		N/A	
Severe-asthma	0/5 (0.0%)	2/124 (1.6%)	N/A		N/A		N/A	
**COPD**								
Non-COPD	4/05 (80.0%)	115/124 (92.7%)	1		1		1	
Mild-COPD	0/05 (0.0%)	8/124 (6.5%)	N/A		N/A		N/A	
Severe-COPD	1/05 (20.0%)	1/124 (0.8%)	28.75 (1.51-546.90)	0.025*	N/A		N/A	
**Normal weight (n = 1,458)**								
**Asthma**								
Non-asthma	90/103 (87.4%)	1,249/1,355 (92.2%)	1		1		1	
Mild-asthma	12/103 (11.7%)	94/1,355 (6.9%)	1.77 (0.94-3.35)	0.079	1.62 (0.78-3.38)	0.197	1.78 (0.83-3.84)	0.142

Severe-asthma	1/103 (1.0%)	12/1,355 (0.9%)	1.16 (0.15-8.99)	0.890	0.28 (0.02-3.18)	0.302	0.33 (0.03-3.84)	0.372
**COPD**								
Non-COPD	95/103 (92.2%)	1,302/1,355 (96.1%)	1		1		1	
Mild-COPD	4/103 (3.9%)	30/1,355 (2.2%)	1.83 (0.63-5.30)	0.267	0.64 (0.18-2.25)	0.482	0.56 (0.15-2.05)	0.379
Severe-COPD	4/103 (3.9%)	23/1,355 (1.7%)	2.39 (0.81-7.04)	0.115	1.05 (0.28-3.98)	0.948	1.07 (0.27-4.26)	0.927
**Overweight (n = 1,010)**								
**Asthma**								
Non-asthma	66/70 (94.3%)	857/940 (91.2%)	1		1		1	
Mild-asthma	3/70 (4.3%)	74/940 (7.9%)	0.53 (0.16-1.72)	0.287	0.28 (0.08-1.02)	0.054	0.26 (0.07-1.03)	0.055
Severe-asthma	1/70 (1.4%)	9/940 (1.0%)	1.44 (0.18-11.56)	0.730	0.94 (0.09-10.11)	0.961	0.91 (0.06-13.77)	0.944
**COPD**								
Non-COPD	66/70 (94.3%)	904/940 (96.2%)	1		1		1	
Mild-COPD	2/70 (2.9%)	25/940 (2.7%)	1.10 (0.25-4.73)	0.902	0.60 (0.12-2.96)	0.527	0.67 (0.12-3.66)	0.647
Severe-COPD	2/70 (2.9%)	11/940 (1.2%)	2.49 (0.54-11.47)	0.241	0.95 (0.14-6.35)	0.960	1.53 (0.18-13.13)	0.699
**Obese (n = 1,469)**								
**Asthma**								
Non-asthma	135/165 (81.8%)	1,188/1,304 (91.1%)	1		1		1	
Mild-asthma	28/165 (17.0%)	103/1,304 (7.9%)	2.39 (1.52-3.77)	<0.001*	1.68 (0.99-2.84)	0.056	1.56 (0.90-2.69)	0.115
Severe-asthma	2/165 (1.2%)	13/1,304 (1.0%)	1.35 (0.30-6.06)	0.692	0.97 (0.17-5.56)	0.971	0.84 (0.13-5.37)	0.857
**COPD**								
Non-COPD	148/165 (89.7%)	1268/1,304 (97.2%)	1		1		1	
Mild-COPD	9/165 (5.5%)	23/1,304 (1.8%)	3.35 (1.52-7.38)	0.003*	1.87 (0.72-4.83)	0.199	1.66 (0.62-4.45)	0.317
Severe-COPD	8/165 (4.8%)	13/1,304 (1.0%)	5.28 (2.15-12.94)	<0.001*	4.98 (1.66-14.93)	0.004*	4.79 (1.57-14.62)	0.006*
**Non-smoker (n = 3,181)**								
**Asthma**								
Non-asthma	203/233 (87.1%)	2,703/2,948 (91.7%)	1		1		1	
Mild-asthma	27/233 (11.6%)	220/2,948 (7.5%)	1.63 (1.07-2.50)	0.023*	1.17 (0.73-1.89)	0.511	1.14 (0.69-1.87)	0.612

Severe-asthma	3/233 (1.3%)	25/2,948 (0.8%)	1.60 (0.48-5.34)	0.446	1.08 (0.27-4.37)	0.919	1.19 (0.30-4.78)	0.803
**COPD**								
Non-COPD	215/233 (92.3%)	2,847/2,948 (96.6%)	1		1		1	
Mild-COPD	7/233 (3.0%)	65/2,948 (2.2%)	1.43 (0.65-3.15)	0.380	0.69 (0.29-1.69)	0.421	0.66 (0.26-1.65)	0.370
Severe-COPD	11/233 (4.7%)	36/2,948 (1.2%)	4.05 (2.03-8.06)	<0.001*	2.55 (1.12-5.85)	0.027*	2.46 (1.06-5.70)	0.037*
**Past or current smoker (n = 885)**								
**Asthma**								
Non-asthma	93/110 (84.5%)	705/775 (91.0%)	1		1		1	
Mild-asthma	16/110 (14.5%)	59/775 (7.6%)	2.06 (1.14-3.72)	0.017*	0.98 (0.47-2.04)	0.958	0.83 (0.38-1.82)	0.649
Severe-asthma	1/110 (0.9%)	11/775 (1.4%)	0.69 (0.09-5.40)	0.723	0.17 (0.02-1.93)	0.154	0.10 (0.01-1.34)	0.082
**COPD**								
Non-COPD	98/110 (89.1%)	742/775 (95.7%)	1		1		1	
Mild-COPD	8/110 (7.3%)	21/775 (2.7%)	2.89 (1.24-6.69)	0.014*	1.66 (0.57-4.83)	0.352	2.06 (0.66-6.37)	0.211
Severe-COPD	4/110 (3.6%)	12/775 (1.5%)	2.52 (0.80-7.98)	0.115	1.21 (0.29-5.08)	0.796	2.06 (0.46-9.35)	0.348
**Alcohol consumption < 1 time a week (n = 2,968)**								
**Asthma**								
Non-asthma	217/258 (84.1%)	2,459/2,710 (90.7%)	1		1		1	
Mild-asthma	38/258 (14.7%)	221/2,710 (8.2%)	1.95 (1.34-2.82)	<0.001*	1.25 (0.81-1.93)	0.321	1.19 (0.76-1.88)	0.447
Severe-asthma	3/258 (1.2%)	30/2,710 (1.1%)	1.13 (0.34-3.74)	0.838	0.42 (0.10-1.73)	0.230	0.38 (0.09-1.64)	0.193
**COPD**								
Non-COPD	234/258 (90.7%)	2,608/2,710 (96.2%)	1		1		1	
Mild-COPD	11/258 (4.3%)	67/2,710 (2.5%)	1.83 (0.95-3.51)	0.069	0.85 (0.39-1.83)	0.671	0.91 (0.41-2.01)	0.817
Severe-COPD	13/258 (5.0%)	35/2,710 (1.3%)	4.14 (2.16-7.93)	<0.001*	2.32 (1.04-5.18)	0.040*	2.38 (1.05-5.38)	0.037*
**Alcohol consumption ≥1 time a week (n = 1,098)**								
**Asthma**								
Non-asthma	79/85 (92.9%)	949/1,013 (93.7%)	1		1		1	
Mild-asthma	5/85 (5.9%)	58/1,013 (5.7%)	1.04 (0.40-2.66)	0.942	0.63 (0.22-1.78)	0.380	0.51 (0.17-1.54)	0.233

Severe-asthma	1/85 (1.2%)	6/1,013 (0.6%)	2.00 (0.24-16.84)	0.523	2.72 (0.28-26.22)	0.387	1.71 (0.15-19.84)	0.668
**COPD**								
Non-COPD	79/85 (92.9%)	981/1,013 (96.8%)	1		1		1	
Mild-COPD	4/85 (4.7%)	19/1,013 (1.9%)	2.62 (0.87-7.88)	0.087	1.87 (0.56-6.28)	0.311	2.31 (0.63-8.52)	0.208
Severe-COPD	2/85 (2.4%)	13/1,013 (1.3%)	1.91 (0.42-8.62)	0.400	1.80 (0.34-9.36)	0.487	1.81 (0.33-9.91)	0.497
**Systolic blood pressure < 140 mmHg and diastolic blood pressure < 90 mmHg (n = 3,530)**								
**Asthma**								
Non-asthma	231/268 (86.2%)	2,987/3,262 (91.6%)	1		1		1	
Mild-asthma	34/268 (12.7%)	246/3,262 (7.5%)	1.79 (1.22-2.62)	0.003*	1.13 (0.73-1.76)	0.587	1.08 (0.68-1.72)	0.732
Severe-asthma	3/268 (1.1%)	29/3,262 (0.9%)	1.34 (0.40-4.42)	0.634	0.37 (0.08-1.68)	0.198	0.32 (0.07-1.56)	0.158
**COPD**								
Non-COPD	246/268 (91.8%)	3,149/3,262 (96.5%)	1		1		1	
Mild-COPD	12/268 (4.5%)	69/3,262 (2.1%)	2.23 (1.19-4.17)	0.012*	1.10 (0.52-2.30)	0.804	1.20 (0.56-2.56)	0.633
Severe-COPD	10/268 (3.7%)	44/3,262 (1.3%)	2.91 (1.45-5.85)	0.003*	1.24 (0.53-2.91)	0.615	1.34 (0.57-3.17)	0.506
**Systolic blood pressure ≥ 140 mmHg or diastolic blood pressure ≥90 mmHg (n = 536)**								
**Asthma**								
Non-asthma	65/75 (86.7%)	421/461 (91.3%)	1		1		1	
Mild-asthma	9/75 (12.0%)	33/461 (7.2%)	1.77 (0.81-3.86)	0.154	1.07 (0.42-2.77)	0.886	1.15 (0.42-3.16)	0.791
Severe-asthma	1/75 (1.3%)	7/461 (1.5%)	0.93 (0.11-7.64)	0.943	1.59 (0.15-17.24)	0.704	1.09 (0.09-13.52)	0.950
**COPD**								
Non-COPD	67/75 (89.3%)	440/461 (95.4%)	1		1		1	
Mild-COPD	3/75 (4.0%)	17/461 (3.7%)	1.16 (0.33-4.06)	0.818	0.72 (0.15-3.39)	0.681	0.68 (0.14-3.43)	0.642
Severe-COPD	5/75 (6.7%)	4/461 (0.9%)	8.21 (2.15-31.34)	0.002*	22.32 (3.28-152.00)	0.002*	22.24 (3.11-158.94)	0.002*
**Fasting blood glucose < 100 mg/dL (n = 2,522)**								
**Asthma**								
Non-asthma	130/153 (85.0%)	2,179/2,369 (92.0%)	1		1		1	
Mild-asthma	21/153 (13.7%)	171/2,369 (7.2%)	2.06 (1.27-3.35)	0.004*	1.18 (0.67-2.09)	0.561	1.09 (0.60-1.96)	0.785

Severe-asthma	2/153 (1.3%)	19/2,369 (0.8%)	1.76 (0.41-7.66)	0.448	1.01 (0.19-5.28)	0.990	0.76 (0.13-4.53)	0.765
**COPD**								
Non-COPD	139/153 (90.8%)	2,285/2,369 (96.5%)	1		1		1	
Mild-COPD	7/153 (4.6%)	51/2,369 (2.2%)	2.26 (1.01-5.06)	0.049*	1.32 (0.52-3.35)	0.564	1.29 (0.50-3.36)	0.596
Severe-COPD	7/153 (4.6%)	33/2,369 (1.4%)	3.49 (1.52-8.02)	0.003*	1.92 (0.68-5.41)	0.218	1.95 (0.66-5.78)	0.229
**Fasting blood glucose ≥ 100 mg/dL (n = 1,544)**								
**Asthma**								
Non-asthma	166/190 (87.4%)	1,229/1,354 (90.8%)	1		1		1	
Mild-asthma	22/190 (11.6%)	108/1,354 (8.0%)	1.51 (0.93-2.45)	0.098	1.03 (0.58-1.80)	0.931	1.01 (0.56-1.81)	0.977
Severe-asthma	2/190 (1.1%)	17/1,354 (1.3%)	0.87 (0.20-3.80)	0.854	0.30 (0.05-1.85)	0.194	0.30 (0.05-2.01)	0.215
**COPD**								
Non-COPD	174/190 (91.6%)	1,304/1,354 (96.3%)	1		1		1	
Mild-COPD	8/190 (4.2%)	35/1,354 (2.6%)	1.71 (0.78-3.75)	0.179	0.80 (0.32-1.99)	0.624	0.97 (0.37-2.53)	0.950
Severe-COPD	8/190 (4.2%)	15/1,354 (1.1%)	4.00 (1.67-9.57)	0.002*	2.15 (0.77-6.03)	0.146	2.23 (0.78-6.31)	0.133
**Total cholesterol < 200 mg/dL (n = 2,325)**								
**Asthma**								
Non-asthma	186/220 (84.5%)	1,923/2,105 (91.4%)	1		1		1	
Mild-asthma	32/220 (14.5%)	158/2,105 (7.5%)	2.09 (1.39-3.15)	<0.001*	1.40 (0.87-2.25)	0.169	1.34 (0.82-2.21)	0.245
Severe-asthma	2/220 (0.9%)	24/2,105 (1.1%)	0.86 (0.20-3.67)	0.840	0.32 (0.06-1.68)	0.177	0.27 (0.05-1.56)	0.142
**COPD**								
Non-COPD	198/220 (90.0%)	2,026/2,105 (96.2%)	1		1		1	
Mild-COPD	10/220 (4.5%)	53/2,105 (2.5%)	1.93 (0.97-3.85)	0.062	0.84 (0.37-1.88)	0.666	0.87 (0.38-2.03)	0.754
Severe-COPD	12/220 (5.5%)	26/2,105 (1.2%)	4.72 (2.35-9.51)	<0.001*	2.45 (1.02-5.85)	0.045*	2.53 (1.04-6.14)	0.040*
**Total cholesterol ≥ 200 mg/dL (n = 1,741)**								
**Asthma**								
Non-asthma	110/123 (89.4%)	1,485/1,618 (91.8%)	1		1		1	
Mild-asthma	11/123 (8.9%)	121/1,618 (7.5%)	1.23 (0.64-2.34)	0.535	0.76 (0.36-1.60)	0.465	0.72 (0.33-1.54)	0.393

Severe-asthma	2/123 (1.6%)	12/1,618 (0.7%)	2.25 (0.50-10.18)	0.292	1.59 (0.23-11.01)	0.637	1.36 (0.18-10.16)	0.765
**COPD**								
Non-COPD	115/123 (93.5%)	1,563/1,618 (96.6%)	1		1		1	
Mild-COPD	5/123 (4.1%)	33/1,618 (2.0%)	2.06 (0.79-5.38)	0.140	1.25 (0.39-3.95)	0.710	1.32 (0.41-4.28)	0.643
Severe-COPD	3/123 (2.4%)	22/1,618 (1.4%)	1.85 (0.55-6.28)	0.322	1.55 (0.40-5.96)	0.525	1.61 (0.41-6.36)	0.497
**CCI scores = 0 (n = 3,089)**								
**Asthma**								
Non-asthma	143/164 (87.2%)	2,697/2,925 (92.2%)	1		1		1	
Mild-asthma	21/164 (12.8%)	198/2,925 (6.8%)	2.00 (1.24-3.23)	0.005*	1.49 (0.87-2.56)	0.144	1.43 (0.82-2.49)	0.204
Severe-asthma	0/164 (0.0%)	30/2,925 (1.0%)	N/A		N/A		N/A	
**COPD**								
Non-COPD	153/164 (93.3%)	2,837/2,925 (97.0%)	1		1		1	
Mild-COPD	4/164 (2.4%)	54/2,925 (1.8%)	1.37 (0.49-3.84)	0.545	1.06 (0.34-3.24)	0.925	0.99 (0.32-3.12)	0.988
Severe-COPD	7/164 (4.3%)	34/2,925 (1.2%)	3.82 (1.67-8.75)	0.002*	2.17 (0.78-6.08)	0.140	2.74 (0.98-7.63)	0.054
**CCI scores = 1 (n = 588)**								
**Asthma**								
Non-asthma	77/91 (84.6%)	447/497 (89.9%)	1		1		1	
Mild-asthma	12/91 (13.2%)	45/497 (9.1%)	1.55 (0.78-3.06)	0.209	0.95 (0.45-2.03)	0.255	0.88 (0.39-1.97)	0.279
Severe-asthma	2/91 (2.2%)	5/497 (1.0%)	2.32 (0.44-12.18)	0.319	2.20 (0.35-13.76)	0.903	1.84 (0.28-12.23)	0.758
**COPD**								
Non-COPD	85/91 (93.4%)	470/497 (94.6%)	1		1		1	
Mild-COPD	3/91 (3.3%)	19/497 (3.8%)	0.87 (0.25-3.02)	0.830	0.80 (0.20-3.18)	0.255	0.77 (0.18-3.37)	0.528
Severe-COPD	3/91 (3.3%)	8/497 (1.6%)	2.07 (0.54-7.97)	0.289	4.06 (0.83-19.80)	0.750	3.89 (0.75-20.26)	0.733
**CCI scores ≥ 2 (n = 389)**								
**Asthma**								
Non-asthma	76/88 (86.4%)	264/301 (87.7%)	1		1		1	
Mild-asthma	10/88 (11.4%)	36/301 (12.0%)	0.97 (0.46-2.03)	0.925	0.48 (0.18-1.29)	0.933	0.46 (0.17-1.28)	0.997

Severe-asthma	2/88 (2.3%)	1/301 (0.3%)	6.94 (0.62-77.51)	0.116	1.17 (0.03-45.02)	0.015*	1.22 (0.03-56.33)	0.015*
**COPD**								
Non-COPD	125/135 (92.6%)	1,746/1,837 (95.0%)	1		1		1	
Mild-COPD	10/135 (7.4%)	86/1,837 (4.7%)	1.62 (0.82-3.20)	0.162	1.03 (0.47-2.27)	0.937	0.88 (0.37-2.07)	0.772
Severe-COPD	0/135 (0.0%)	5/1,837 (0.3%)	N/A		N/A		N/A	
**NSAID used < 6 times (n = 1,972)**								
**Asthma**								
Non-asthma	125/135 (92.6%)	1,746/1,837 (95.0%)	1		1		1	
Mild-asthma	10/135 (7.4%)	86/1,837 (4.7%)	1.62 (0.82-3.20)	0.162	1.03 (0.47-2.27)	0.937	0.88 (0.37-2.07)	0.772
Severe-asthma	0/135 (0.0%)	5/1,837 (0.3%)	N/A		N/A		N/A	
**COPD**								
Non-COPD	124/135 (91.9%)	1,788/1,837 (97.3%)	1		1		1	
Mild-COPD	4/135 (3.0%)	31/1,837 (1.7%)	1.86 (0.65-5.36)	0.250	0.90 (0.28-2.89)	0.860	0.99 (0.29-3.38)	0.990
Severe-COPD	7/135 (5.2%)	18/1,837 (1.0%)	5.61 (2.30-13.68)	<0.001*	4.93 (1.68-14.44)	0.004*	5.05 (1.71-14.95)	0.003*
**NSAID used ≥ 6 times (n = 2,094)**								
**Asthma**								
Non-asthma	171/208 (82.2%)	1,662/1,886 (88.1%)	1		1		1	
Mild-asthma	33/208 (15.9%)	193/1,886 (10.2%)	1.66 (1.11-2.48)	0.013*	1.11 (0.70-1.77)	0.660	1.11 (0.69-1.78)	0.683
Severe-asthma	4/208 (1.9%)	31/1,886 (1.6%)	1.25 (0.44-3.60)	0.674	0.56 (0.15-2.05)	0.379	0.54 (0.14-2.09)	0.375
**COPD**								
Non-COPD	189/208 (90.9%)	1,801/1,886 (95.5%)	1		1		1	
Mild-COPD	11/208 (5.3%)	55/1,886 (2.9%)	1.91 (0.98-3.70)	0.057	0.94 (0.41-2.12)	0.872	0.98 (0.42-2.27)	0.964
Severe-COPD	8/208 (3.8%)	30/1,886 (1.6%)	2.54 (1.15-5.62)	0.021*	1.11 (0.41-2.99)	0.831	1.18 (0.43-3.24)	0.745
**Steroid used < 3 times (n = 2,206)**								
**Asthma**								
Non-asthma	138/144 (95.8%)	1,963/2,062 (95.2%)	1		1		1	
Mild-asthma	6/144 (4.2%)	92/2,062 (4.5%)	0.93 (0.40-2.16)	0.862	0.67 (0.27-1.66)	0.391	0.62 (0.24-1.59)	0.319

Severe-asthma	0/144 (0.0%)	07/2,062 (0.3%)	N/A		N/A		N/A	
**COPD**								
Non-COPD	139/144 (96.5%)	2,025/2,062 (98.2%)	1		1		1	
Mild-COPD	3/144 (2.1%)	28/2,062 (1.4%)	1.56 (0.47-5.20)	0.468	1.41 (0.38-5.17)	0.605	1.59 (0.42-5.99)	0.492
Severe-COPD	2/144 (1.4%)	9/2,062 (0.4%)	3.24 (0.69-15.13)	0.135	1.19 (0.19-7.40)	0.853	1.53 (0.23-9.99)	0.660
**Steroid used ≥ 3 times (n = 1,860)**								
**Asthma**								
Non-asthma	158/199 (79.4%)	1,445/1,661 (87.0%)	1		1		1	
Mild-asthma	37/199 (18.6%)	187/1,661 (11.3%)	1.81 (1.23-2.67)	0.003*	1.34 (0.85-2.13)	0.213	1.31 (0.82-2.11)	0.262
Severe-asthma	4/199 (2.0%)	29/1,661 (1.7%)	1.26 (0.44-3.64)	0.667	0.51 (0.14-1.83)	0.300	0.45 (0.12-1.70)	0.238
**COPD**								
Non-COPD	174/199 (87.4%)	1,564/1,661 (94.2%)	1		1		1	
Mild-COPD	12/199 (6.0%)	58/1,661 (3.5%)	1.86 (0.98-3.53)	0.619	0.82 (0.38-1.79)	0.376	0.84 (0.38-1.89)	0.678
Severe-COPD	13/199 (6.5%)	39/1,661 (2.3%)	3.00 (1.57-5.72)	0.001*	2.52 (1.14-5.54)	0.022*	2.61 (1.17-5.81)	0.019*
**Non-hypertension (n = 2,878)**								
**Asthma**								
Non-asthma	147/165 (89.1%)	2,515/2,713 (92.7%)	1		1		1	
Mild-asthma	18/165 (10.9%)	176/2,713 (6.5%)	1.75 (1.05-2.92)	0.033	1.23 (0.69-2.19)	0.492	1.26 (0.69-2.29)	0.447
Severe-asthma	0/165 (0.0%)	22/2,713 (0.8%)	N/A		N/A		N/A	
**COPD**								
Non-COPD	157/165 (95.2%)	2,627/2,713 (96.8%)	1		1		1	
Mild-COPD	5/165 (3.0%)	50/2,713 (1.8%)	1.67 (0.66-4.26)	0.280	1.04 (0.36-2.96)	0.946	0.95 (0.32-2.77)	0.919
Severe-COPD	3/165 (1.8%)	36/2,713 (1.3%)	1.39 (0.43-4.58)	0.584	0.59 (0.14-2.40)	0.459	0.65 (0.15-2.78)	0.556
**Hypertension (n = 1,188)**								
**Asthma**								
Non-asthma	149/178 (83.7%)	893/1,010 (88.4%)	1		1		1	
Mild-asthma	25/178 (14.0%)	103/1,010 (10.2%)	1.46 (0.91-2.33)	0.118	1.03 (0.60-1.76)	0.930	0.92 (0.52-1.62)	0.764

Severe-asthma	4/178 (2.2%)	14/1,010 (1.4%)	1.71 (0.56-5.27)	0.349	0.90 (0.24-3.40)	0.881	0.72 (0.17-2.99)	0.650
**COPD**								
Non-COPD	156/178 (87.6%)	962/1,010 (95.2%)	1		1		1	
Mild-COPD	10/178 (5.6%)	36/1,010 (3.6%)	1.71 (0.83-3.52)	0.143	0.87 (0.38-2.00)	0.746	0.95 (0.39-2.29)	0.904
Severe-COPD	12/178 (6.7%)	12/1,010 (1.2%)	6.17 (2.72-13.97)	<0.001*	4.36 (1.66-11.43)	0.003*	4.63 (1.71-12.54)	0.003*

* Unconditional logistic regression model, Significance at P < 0.05

† Model 1 was adjusted for age, sex, income, obesity, smoking, alcohol consumption, systolic blood pressure, diastolic blood pressure, fasting blood glucose, total cholesterol, CCI scores, number of NSAID used, number of steroid used and hypertension.

‡ Model 2 was adjusted for model 1 plus asthma and COPD.

Table S3. Subgroup analyses of crude and adjusted odd ratios of asthma and COPD for mortality in COVID-19 participants by covariates

Characteristics	Dead participants (exposure/total, %)	Survived participants (exposure/total, %)	ORs (95% confidence interval) for mortality					
			Crude	P-value	Model 1†	P-value	Model 2‡	P-value
**Age < 60 years old (n = 2,486)**								
**Asthma**								
Non-asthma	12/12 (100.0%)	2,305/2,474 (93.2%)	1		1		1	
Mild-asthma	0/12 (0.0%)	151/2,474 (6.1%)	N/A		N/A		N/A	
Severe-asthma	0/12 (0.0%)	18/2,474 (0.7%)	N/A		N/A		N/A	
**COPD**								
Non-COPD	11/12 (91.7%)	2,413/2,474 (97.5%)	1		1		1	
Mild-COPD	0/12 (0.0%)	34/2,474 (1.4%)	N/A		N/A		N/A	
Severe-COPD	1/12 (8.3%)	27/2,474 (1.1%)	8.13 (1.01-65.16)	0.049*	13.09 (0.87-196.11)	0.063	32.84 (2.43-444.17)	0.009*
**Age ≥ 60 years old (n = 1,580)**								
**Asthma**								
Non-asthma	96/120 (80.0%)	1,291/1,460 (88.4%)	1		1		1	
Mild-asthma	20/120 (16.7%)	151/1,460 (10.3%)	1.78 (1.07-2.97)	0.027*	1.07 (0.58-1.97)	0.837	0.93 (0.49-1.79)	0.838
Severe-asthma	4/120 (3.3%)	18/1,460 (1.2%)	2.99 (0.99-9.01)	0.052	1.10 (0.23-5.25)	0.910	0.73 (0.13-4.08)	0.722
**COPD**								
Non-COPD	102/120 (85.0%)	1,376/1,460 (94.2%)	1		1		1	
Mild-COPD	9/120 (7.5%)	58/1,460 (4.0%)	2.09 (1.01-4.35)	0.047*	1.13 (0.45-2.85)	0.794	1.20 (0.45-3.19)	0.710
Severe-COPD	9/120 (7.5%)	26/1,460 (1.8%)	4.67 (2.13-10.23)	0.001*	2.73 (1.01-7.41)	0.049*	2.90 (1.02-8.26)	0.047*
**Men (n = 1,526)**								
**Asthma**								
Non-asthma	74/91 (81.3%)	1,332/1,435 (92.8%)	1		1		1	
Mild-asthma	15/91 (16.5%)	91/1,435 (6.3%)	2.97 (1.64-5.38)	<0.001*	1.22 (0.58-2.56)	0.605	0.97 (0.44-2.15)	0.945

Severe-asthma	2/91 (2.2%)	12/1,435 (0.8%)	3.00 (0.66-13.65)	0.155	0.27 (0.03-2.90)	0.282	0.13 (0.01-1.71)	0.121
**COPD**								
Non-COPD	76/91 (83.5%)	1,381/1,435 (96.2%)	1		1		1	
Mild-COPD	9/91 (9.9%)	30/1,435 (2.1%)	5.45 (2.50-11.89)	<0.001*	2.30 (0.78-6.81)	0.133	2.67 (0.86-8.27)	0.090*
Severe-COPD	6/91 (6.6%)	24/1,435 (1.7%)	4.54 (1.80-11.44)	0.001*	1.57 (0.47-5.20)	0.461	2.14 (0.61-7.51)	0.237
**Women (n = 2,540)**								
**Asthma**								
Non-asthma	34/41 (82.9%)	2,264/2,499 (90.6%)	1		1		1	
Mild-asthma	5/41 (12.2%)	211/2,499 (8.4%)	1.58 (0.61-4.08)	0.346	0.52 (0.17-1.61)	0.258	0.39 (0.11-1.42)	0.155
Severe-asthma	2/41 (4.9%)	24/2,499 (1.0%)	5.55 (1.26-24.42)	0.023*	2.52 (0.40-15.97)	0.326	5.78 (0.82-40.67)	0.078
**COPD**								
Non-COPD	37/41 (90.2%)	2,408/2,499 (96.4%)	1		1		1	
Mild-COPD	0/41 (0.0%)	62/2,499 (2.5%)	N/A		N/A		N/A	
Severe-COPD	4/41 (9.8%)	29/2,499 (1.2%)	8.98 (3.00-26.82)	<0.001*	5.74 (1.34-24.63)	0.019*	8.26 (1.63-41.81)	0.011*
**Low income (n = 1,472)**								
**Asthma**								
Non-asthma	30/35 (85.7%)	1,313/1,437 (91.4%)	1		1		1	
Mild-asthma	4/35 (11.4%)	112/1,437 (7.8%)	1.56 (0.54-4.52)	0.409	0.56 (0.15-2.15)	0.397	0.61 (0.15-2.41)	0.479
Severe-asthma	1/35 (2.9%)	12/1,437 (0.8%)	3.65 (0.46-28.96)	0.221	11.09 (1.06-116.04)	0.045*	11.35 (0.69-187.10)	0.089
**COPD**								
Non-COPD	30/35 (85.7%)	1,385/1,437 (96.4%)	1		1		1	
Mild-COPD	1/35 (2.9%)	35/1,437 (2.4%)	1.32 (0.18-9.95)	0.788	0.24 (0.02-3.47)	0.297	0.23 (0.02-3.18)	0.272
Severe-COPD	4/35 (11.4%)	17/1,437 (1.2%)	10.87 (3.45-34.24)	<0.001*	6.75 (1.26-36.05)	0.026*	5.15 (0.90-29.67)	0.066
**Middle income (n = 1,249)**								
**Asthma**								
Non-asthma	31/40 (77.5%)	1,111/1,209 (91.9%)	1		1		1	
Mild-asthma	7/40 (17.5%)	87/1,209 (7.2%)	2.88 (1.23-6.74)	0.015*	1.15 (0.32-4.19)	0.834	1.22 (0.32-4.65)	0.771

Severe-asthma	2/40 (5.0%)	11/1,209 (0.9%)	6.52 (1.39-30.65)	0.017*	0.09 (0.01-1.46)	0.091	0.11 (0.00-3.49)	0.211
**COPD**								
Non-COPD	35/40 (87.5%)	1,171/1,209 (96.9%)	1		1		1	
Mild-COPD	3/40 (7.5%)	23/1,209 (1.9%)	4.36 (1.25-15.22)	0.021*	0.36 (0.03-3.88)	0.397	0.89 (0.07-11.12)	0.927
Severe-COPD	2/40 (5.0%)	15/1,209 (1.2%)	4.46 (0.98-20.26)	0.053	0.34 (0.02-5.40)	0.444	0.61 (0.03-12.08)	0.745
**High income (n = 1,345)**								
**Asthma**								
Non-asthma	47/57 (82.5%)	1,172/1,288 (91.0%)	1		1		1	
Mild-asthma	9/57 (15.8%)	103/1,288 (8.0%)	2.18 (1.04-4.57)	0.039*	1.19 (0.48-2.96)	0.705	0.97 (0.37-2.54)	0.944
Severe-asthma	1/57 (1.8%)	13/1,288 (1.0%)	1.92 (0.25-14.97)	0.534	1.05 (0.10-11.55)	0.968	0.60 (0.04-9.09)	0.711
**COPD**								
Non-COPD	48/57 (84.2%)	1,233/1,288 (95.7%)	1		1		1	
Mild-COPD	5/57 (8.8%)	34/1,288 (2.6%)	3.78 (1.42-10.09)	0.008*	1.80 (0.54-6.00)	0.337	1.85 (0.53-6.51)	0.335
Severe-COPD	4/57 (7.0%)	21/1,288 (1.6%)	4.89 (1.62-14.81)	0.005*	3.02 (0.70-13.01)	0.139	3.24 (0.71-14.73)	0.128
**Underweight (n = 129)**								
**Asthma**								
Non-asthma	3/3 (100.0%)	116/126 (92.1%)	1		1		1	
Mild-asthma	0/3 (0.0%)	8/126 (6.3%)	N/A		N/A		N/A	
Severe-asthma	0/3 (0.0%)	2/126 (1.6%)	N/A		N/A		N/A	
**COPD**								
Non-COPD	2/03 (66.7%)	117/126 (92.9%)	1		1		1	
Mild-COPD	0/03 (0.0%)	8/126 (6.3%)	N/A		N/A		N/A	
Severe-COPD	1/03 (33.3%)	1/126 (0.8%)	N/A		N/A		N/A	
**Normal weight (n = 1,458)**								
**Asthma**								
Non-asthma	33/37 (89.2%)	1,306/1,421 (91.9%)	1		1		1	
Mild-asthma	3/37 (8.1%)	103/1,421 (7.2%)	1.15 (0.35-3.82)	0.816	0.74 (0.16-3.38)	0.701	1.00 (0.19-5.27)	0.996

Severe-asthma	1/37 (2.7%)	12/1,421 (0.8%)	3.30 (0.42-26.11)	0.258	0.06 (0.00-3.23)	0.169	0.10 (0.00-4.00)	0.220
**COPD**								
Non-COPD	33/37 (89.2%)	1,364/1,421 (96.0%)	1		1		1	
Mild-COPD	2/37 (5.4%)	32/1,421 (2.3%)	2.58 (0.59-11.23)	0.206	0.20 (0.02-2.07)	0.178	0.27 (0.03-2.61)	0.255
Severe-COPD	2/37 (5.4%)	25/1,421 (1.8%)	3.31 (0.75-14.54)	0.113	0.66 (0.10-4.49)	0.667	1.21 (0.15-9.96)	0.861
**Overweight (n = 1,010)**								
**Asthma**								
Non-asthma	27/28 (96.4%)	896/982 (91.2%)	1		1		1	
Mild-asthma	0/28 (0.0%)	77/982 (7.8%)	N/A		N/A		N/A	
Severe-asthma	1/28 (3.6%)	9/982 (0.9%)	3.69 (0.45-30.15)	0.224	2.93 (0.19-45.86)	0.444	N/A	
**COPD**								
Non-COPD	25/28 (89.3%)	945/982 (96.2%)	1		1		1	
Mild-COPD	1/28 (3.6%)	26/982 (2.6%)	1.45 (0.19-11.14)	0.719	0.61 (0.05-6.93)	0.692	0.99 (0.06-16.10)	0.997
Severe-COPD	2/28 (7.1%)	11/982 (1.1%)	6.87 (1.45-32.64)	0.015*	3.22 (0.36-29.19)	0.298	N/A	
**Obese (n = 1,469)**								
**Asthma**								
Non-asthma	45/64 (70.3%)	1,278/1,405 (91.0%)	1		1		1	
Mild-asthma	17/64 (26.6%)	114/1,405 (8.1%)	4.24 (2.35-7.64)	<0.001*	2.51 (1.20-5.28)	0.015*	2.24 (1.03-4.88)	0.042*
Severe-asthma	2/64 (3.1%)	13/1,405 (0.9%)	4.37 (0.96-19.94)	0.057	3.35 (0.37-30.04)	0.280	3.02 (0.30-31.00)	0.352
**COPD**								
Non-COPD	53/64 (82.8%)	1,363/1,405 (97.0%)	1		1		1	
Mild-COPD	6/64 (9.4%)	26/1,405 (1.9%)	5.94 (2.34-15.03)	<0.001*	2.37 (0.66-8.45)	0.185	1.62 (0.41-6.39)	0.492
Severe-COPD	5/64 (7.8%)	16/1,405 (1.1%)	8.04 (2.84-22.76)	<0.001*	7.07 (1.61-31.04)	0.010*	5.96 (1.31-27.02)	0.021*
**Non-smoker (n = 3,181)**								
**Asthma**								
Non-asthma	65/77 (84.4%)	2,841/3,104 (91.5%)	1		1		1	
Mild-asthma	9/77 (11.7%)	238/3,104 (7.7%)	1.65 (0.81-3.36)	0.165	0.74 (0.32-1.70)	0.475	0.70 (0.29-1.69)	0.427

Severe-asthma	3/77 (3.9%)	25/3,104 (0.8%)	5.25 (1.55-17.81)	0.008*	3.67 (0.68-19.96)	0.132	4.61 (0.84-25.15)	0.078
**COPD**								
Non-COPD	67/77 (87.0%)	2,995/3,104 (96.5%)	1		1		1	
Mild-COPD	3/77 (3.9%)	69/3,104 (2.2%)	1.95 (0.60-6.33)	0.269	0.48 (0.11-2.08)	0.329	0.38 (0.07-1.96)	0.246
Severe-COPD	7/77 (9.1%)	40/3,104 (1.3%)	7.82 (3.38-18.10)	<0.001*	3.32 (1.04-10.56)	0.042*	3.53 (1.06-11.76)	0.040*
**Past or current smoker (n = 885)**								
**Asthma**								
Non-asthma	43/55 (78.2%)	755/830 (91.0%)	1		1		1	
Mild-asthma	11/55 (20.0%)	64/830 (7.7%)	3.02 (1.48-6.14)	0.002*	1.14 (0.44-2.96)	0.783	0.83 (0.29-2.34)	0.717
Severe-asthma	1/55 (1.8%)	11/830 (1.3%)	1.60 (0.20-12.65)	0.658	0.10 (0.01-1.67)	0.108	0.04 (0.00-0.96)	0.047*
**COPD**								
Non-COPD	46/55 (83.6%)	794/830 (95.7%)	1		1		1	
Mild-COPD	6/55 (10.9%)	23/830 (2.8%)	4.50 (1.75-11.60)	0.002*	3.08 (0.76-12.59)	0.117	4.00 (0.90-17.78)	0.068
Severe-COPD	3/55 (5.5%)	13/830 (1.6%)	3.98 (1.10-14.47)	0.036*	0.98 (0.16-5.90)	0.984	2.40 (0.34-17.19)	0.383
**Alcohol consumption < 1 time a week (n = 2,968)**								
**Asthma**								
Non-asthma	92/112 (82.1%)	2,584/2,856 (90.5%)	1		1		1	
Mild-asthma	17/112 (15.2%)	242/2,856 (8.5%)	1.97 (1.16-3.37)	0.013*	0.86 (0.44-1.67)	0.659	0.79 (0.39-1.58)	0.496
Severe-asthma	3/112 (2.7%)	30/2,856 (1.1%)	2.81 (0.84-9.37)	0.093	0.61 (0.11-3.41)	0.572	0.45 (0.07-2.90)	0.404
**COPD**								
Non-COPD	97/112 (86.6%)	2,745/2,856 (96.1%)	1		1		1	
Mild-COPD	7/112 (6.3%)	71/2,856 (2.5%)	2.79 (1.25-6.22)	0.012*	0.87 (0.30-2.51)	0.798	1.06 (0.35-3.19)	0.917
Severe-COPD	8/112 (7.1%)	40/2,856 (1.4%)	5.66 (2.58-12.42)	<0.001*	2.16 (0.76-6.20)	0.151	2.54 (0.85-7.58)	0.096
**Alcohol consumption ≥1 time a week (n = 1,098)**								
**Asthma**								
Non-asthma	16/20 (80.0%)	1,012/1,078 (93.9%)	1		1		1	
Mild-asthma	3/20 (15.0%)	60/1,078 (5.6%)	3.16 (0.90-11.15)	0.073	1.60 (0.29-8.94)	0.590	1.22 (0.20-7.60)	0.832

Severe-asthma	1/20 (5.0%)	6/1,078 (0.6%)	10.54 (1.20-92.68)	0.034*	9.35 (0.43-201.98)	0.154	2.71 (0.08-96.04)	0.584
**COPD**								
Non-COPD	16/20 (80.0%)	1,044/1,078 (96.8%)	1		1		1	
Mild-COPD	2/20 (10.0%)	21/1,078 (1.9%)	6.22 (1.34-28.76)	0.019*	2.63 (0.30-23.21)	0.385	2.21 (0.23-21.62)	0.497
Severe-COPD	2/20 (10.0%)	13/1,078 (1.2%)	10.04 (2.09-48.18)	0.004*	10.24 (0.89-118.09)	0.062	7.35 (0.45-119.08)	0.160
**Systolic blood pressure < 140 mmHg and diastolic blood pressure < 90 mmHg (n = 3,530)**								
**Asthma**								
Non-asthma	82/101 (81.2%)	3,136/3,429 (91.5%)	1		1		1	
Mild-asthma	16/101 (15.8%)	264/3,429 (7.7%)	2.32 (1.34-4.02)	0.003*	0.93 (0.46-1.88)	0.848	0.82 (0.39-1.71)	0.593
Severe-asthma	3/101 (3.0%)	29/3,429 (0.8%)	3.96 (1.18-13.25)	0.026*	0.37 (0.06-2.39)	0.294	0.23 (0.03-1.78)	0.159
**COPD**								
Non-COPD	87/101 (86.1%)	3,308/3,429 (96.5%)	1		1		1	
Mild-COPD	7/101 (6.9%)	74/3,429 (2.2%)	3.60 (1.61-8.04)	0.002*	1.17 (0.39-3.51)	0.780	1.56 (0.50-4.87)	0.443
Severe-COPD	7/101 (6.9%)	47/3,429 (1.4%)	5.66 (2.49-12.89)	<0.001*	1.52 (0.51-4.55)	0.455	1.98 (0.63-6.23)	0.244
**Systolic blood pressure ≥ 140 mmHg or diastolic blood pressure ≥90 mmHg (n = 536)**								
**Asthma**								
Non-asthma	26/31 (83.9%)	460/505 (91.1%)	1		1		1	
Mild-asthma	4/31 (12.9%)	38/505 (7.5%)	1.86 (0.62-5.61)	0.269	1.05 (0.26-4.21)	0.946	1.07 (0.22-5.10)	0.937
Severe-asthma	1/31 (3.2%)	07/505 (1.4%)	2.53 (0.30-21.32)	0.394	5.82 (0.35-96.76)	0.220	4.49 (0.23-89.50)	0.325
**COPD**								
Non-COPD	26/31 (83.9%)	481/505 (95.2%)	1		1		1	
Mild-COPD	2/31 (6.5%)	18/505 (3.6%)	2.06 (0.45-9.34)	0.351	0.95 (0.11-8.13)	0.964	0.77 (0.07-8.19)	0.829
Severe-COPD	3/31 (9.7%)	6/505 (1.2%)	9.25 (2.19-39.08)	0.003*	33.28 (3.52-314.96)	0.002*	28.33 (3.13-256.62)	0.003*
**Fasting blood glucose < 100 mg/dL (n = 2,522)**								
**Asthma**								
Non-asthma	45/56 (80.4%)	2,264/2,466 (91.8%)	1		1		1	
Mild-asthma	9/56 (16.1%)	183/2,466 (7.4%)	2.47 (1.19-5.14)	0.015*	0.88 (0.35-2.20)	0.784	0.77 (0.29-2.04)	0.594

Severe-asthma	2/56 (3.6%)	19/2,466 (0.8%)	5.30 (1.20-23.42)	0.028*	2.69 (0.37-19.65)	0.328	1.60 (0.16-16.53)	0.692
**COPD**								
Non-COPD	48/56 (85.7%)	2376/2,466 (96.4%)	1		1		1	
Mild-COPD	3/56 (5.4%)	55/2,466 (2.2%)	2.70 (0.82-8.93)	0.104	1.00 (0.23-4.38)	0.998	1.06 (0.23-4.78)	0.943
Severe-COPD	5/56 (8.9%)	35/2,466 (1.4%)	7.07 (2.66-18.84)	<0.001*	2.84 (0.70-11.54)	0.146	2.80 (0.57-13.66)	0.203
**Fasting blood glucose ≥ 100 mg/dL (n = 1,544)**								
**Asthma**								
Non-asthma	63/76 (82.9%)	1,332/1,468 (90.7%)	1		1		1	
Mild-asthma	11/76 (14.5%)	119/1,468 (8.1%)	1.95 (1.00-3.81)	0.049*	1.02 (0.42-2.45)	0.967	0.90 (0.36-2.21)	0.809
Severe-asthma	2/76 (2.6%)	17/1,468 (1.2%)	2.49 (0.56-11.01)	0.229	0.36 (0.04-3.66)	0.390	0.27 (0.02-3.55)	0.316
**COPD**								
Non-COPD	65/76 (85.5%)	1,413/1,468 (96.3%)	1		1		1	
Mild-COPD	6/76 (7.9%)	37/1,468 (2.5%)	3.53 (1.44-8.65)	0.006*	1.12 (0.33-3.83)	0.855	1.53 (0.41-5.71)	0.531
Severe-COPD	5/76 (6.6%)	18/1,468 (1.2%)	6.04 (2.17-16.77)	0.001*	3.16 (0.85-11.73)	0.085	3.28 (0.86-12.50)	0.083
**Total cholesterol < 200 mg/dL (n = 2,325)**								
**Asthma**								
Non-asthma	72/91 (79.1%)	2,037/2,234 (91.2%)	1		1		1	
Mild-asthma	17/91 (18.7%)	173/2,234 (7.7%)	2.78 (1.60-4.82)	<0.001*	1.71 (0.84-3.50)	0.139	1.47 (0.68-3.15)	0.324
Severe-asthma	2/91 (2.2%)	24/2,234 (1.1%)	2.36 (0.55-10.17)	0.250	0.48 (0.06-3.75)	0.483	0.25 (0.03-2.34)	0.224
**COPD**								
Non-COPD	75/91 (82.4%)	2,149/2,234 (96.2%)	1		1		1	
Mild-COPD	7/91 (7.7%)	56/2,234 (2.5%)	3.58 (1.58-8.12)	0.002*	1.05 (0.35-3.15)	0.938	1.08 (0.34-3.44)	0.893
Severe-COPD	9/91 (9.9%)	29/2,234 (1.3%)	8.89 (4.07-19.45)	<0.001*	3.98 (1.29-12.29)	0.016*	4.34 (1.33-14.15)	0.015*
**Total cholesterol ≥ 200 mg/dL (n = 1,741)**								
**Asthma**								
Non-asthma	36/41 (87.8%)	1,559/1,700 (91.7%)	1		1		1	
Mild-asthma	3/41 (7.3%)	129/1,700 (7.6%)	1.01 (0.31-3.32)	0.991	0.25 (0.06-1.04)	0.056	0.25 (0.06-1.11)	0.069

Severe-asthma	2/41 (4.9%)	12/1,700 (0.7%)	7.22 (1.56-33.43)	0.012*	5.65 (0.60-53.49)	0.131	5.75 (0.54-61.00)	0.147
**COPD**								
Non-COPD	38/41 (92.7%)	1,640/1,700 (96.5%)	1		1		1	
Mild-COPD	2/41 (4.9%)	36/1,700 (2.1%)	2.40 (0.56-10.32)	0.240	0.66 (0.10-4.47)	0.666	0.64 (0.07-5.93)	0.697
Severe-COPD	1/41 (2.4%)	24/1,700 (1.4%)	1.80 (0.24-13.64)	0.570	0.91 (0.09-8.94)	0.938	1.04 (0.08-12.86)	0.975
**CCI scores = 0 (n = 3,089)**								
**Asthma**								
Non-asthma	36/41 (87.8%)	2,804/3,048 (92.0%)	1		1		1	
Mild-asthma	5/41 (12.2%)	214/3,048 (7.0%)	1.82 (0.71-4.69)	0.215	0.75 (0.24-2.35)	0.621	0.61 (0.17-2.15)	0.442
Severe-asthma	0/41 (0.0%)	30/3,048 (1.0%)	N/A		N/A		N/A	
**COPD**								
Non-COPD	36/41 (87.8%)	2,954/3,048 (96.9%)	1		1		1	
Mild-COPD	1/41 (2.4%)	57/3,048 (1.9%)	1.44 (0.19-10.68)	0.722	0.69 (0.06-8.17)	0.765	0.92 (0.08-10.54)	0.945
Severe-COPD	4/41 (9.8%)	37/3,048 (1.2%)	8.88 (3.01-26.20)	<0.001*	2.20 (0.41-11.87)	0.360	4.53 (0.74-27.70)	0.102
**CCI scores = 1 (n = 588)**								
**Asthma**								
Non-asthma	25/34 (73.5%)	499/554 (90.1%)	1		1		1	
Mild-asthma	7/34 (20.6%)	50/554 (9.0%)	2.79 (1.15-6.79)	0.023*	1.33 (0.49-3.66)	0.016*	1.45 (0.49-4.33)	0.028*
Severe-asthma	2/34 (5.9%)	5/554 (0.9%)	7.99 (1.48-43.20)	0.016*	8.00 (0.95-67.62)	0.576	16.26 (1.62-163.44)	0.506
**COPD**								
Non-COPD	32/34 (94.1%)	523/554 (94.4%)	1		1		1	
Mild-COPD	0/34 (0.0%)	22/554 (4.0%)	N/A		N/A		N/A	
Severe-COPD	2/34 (5.9%)	9/554 (1.6%)	3.63 (0.75-17.51)	0.108	3.31 (0.33-39.91)	0.971	4.11 (0.39-43.38)	0.965
**CCI scores ≥ 2 (n = 389)**								
**Asthma**								
Non-asthma	47/57 (82.5%)	293/332 (88.3%)	1		1		1	
Mild-asthma	8/57 (14.0%)	38/332 (11.4%)	1.31 (0.58-2.99)	0.517	0.48 (0.15-1.58)	0.095	0.42 (0.12-1.44)	0.055

Severe-asthma	2/57 (3.5%)	1/332 (0.3%)	12.46 (1.11-140.11)	0.041*	1.71 (0.04-82.12)	0.098	1.02 (0.02-58.25)	0.065
**COPD**								
Non-COPD	45/57 (78.9%)	312/332 (94.0%)	1		1		1	
Mild-COPD	8/57 (14.0%)	13/332 (3.9%)	4.27 (1.68-10.86)	0.002*	1.50 (0.39-5.79)	0.037*	1.84 (0.41-8.17)	0.170
Severe-COPD	4/57 (7.0%)	7/332 (2.1%)	3.96 (1.12-14.07)	0.038*	3.02 (0.47-19.44)	0.106	3.36 (0.48-23.51)	0.994
**NSAID used < 6 times (n = 1,972)**								
**Asthma**								
Non-asthma	46/48 (95.8%)	1,825/1,924 (94.9%)	1		1		1	
Mild-asthma	2/48 (4.2%)	94/1,924 (4.9%)	0.84 (0.20-3.53)	0.816	0.15 (0.02-1.10)	0.062	0.06 (0.01-0.54)	0.013*
Severe-asthma	0/48 (0.0%)	05/1,924 (0.3%)	N/A		N/A		N/A	
**COPD**								
Non-COPD	42/48 (87.5%)	1,870/1,924 (97.2%)	1		1		1	
Mild-COPD	2/48 (4.2%)	33/1,924 (1.7%)	2.70 (0.63-11.62)	0.183	0.67 (0.12-3.72)	0.648	1.16 (0.17-8.00)	0.879
Severe-COPD	4/48 (8.3%)	21/1,924 (1.1%)	8.48 (2.79-25.79)	<0.001*	6.88 (1.37-34.63)	0.019*	16.84 (3.23-87.87)	0.001*
**NSAID used ≥ 6 times (n = 2,094)**								
**Asthma**								
Non-asthma	62/84 (73.8%)	1,771/2,010 (88.1%)	1		1		1	
Mild-asthma	18/84 (21.4%)	208/2,010 (10.3%)	2.47 (1.44-4.26)	0.001*	1.35 (0.70-2.63)	0.375	1.35 (0.68-2.68)	0.396
Severe-asthma	4/84 (4.8%)	31/2,010 (1.5%)	3.69 (1.26-10.76)	0.017*	1.46 (0.32-6.68)	0.628	1.37 (0.27-6.94)	0.703
**COPD**								
Non-COPD	71/84 (84.5%)	1,919/2,010 (95.5%)	1		1		1	
Mild-COPD	7/84 (8.3%)	59/2,010 (2.9%)	3.21 (1.41-7.27)	0.005*	1.08 (0.36-3.26)	0.890	0.93 (0.29-3.02)	0.904
Severe-COPD	6/84 (7.1%)	32/2,010 (1.6%)	5.07 (2.05-12.51)	<0.001*	1.53 (0.42-5.66)	0.522	1.37 (0.36-5.27)	0.646
**Steroid used < 3 times (n = 2,206)**								
**Asthma**								
Non-asthma	36/36 (100.0%)	2,065/2,170 (95.2%)	1		1		1	
Mild-asthma	0/36 (0.0%)	98/2,170 (4.5%)	N/A		N/A		N/A	

Severe-asthma	0/36 (0.0%)	7/2,170 (0.3%)	N/A		N/A		N/A	
**COPD**								
Non-COPD	34/36 (94.4%)	2,130/2,170 (98.2%)	1		1		1	
Mild-COPD	1/36 (2.8%)	30/2,170 (1.4%)	2.09 (0.28-15.76)	0.475	1.78 (0.14-22.51)	0.657	2.05 (0.14-31.14)	0.604
Severe-COPD	1/36 (2.8%)	10/2,170 (0.5%)	6.27 (0.78-50.31)	0.084	0.55 (0.03-9.29)	0.678	10.53 (0.37-300.82)	0.169
**Steroid used ≥ 3 times (n = 1,860)**								
**Asthma**								
Non-asthma	72/96 (75.0%)	1,531/1,764 (86.8%)	1		1		1	
Mild-asthma	20/96 (20.8%)	204/1,764 (11.6%)	2.09 (1.24-3.49)	0.005*	1.23 (0.64-2.37)	0.543	1.20 (0.60-2.38)	0.606
Severe-asthma	4/96 (4.2%)	29/1,764 (1.6%)	2.93 (1.00-8.57)	0.049*	0.94 (0.21-4.15)	0.938	0.71 (0.14-3.64)	0.681
**COPD**								
Non-COPD	79/96 (82.3%)	1659/1,764 (94.0%)	1		1		1	
Mild-COPD	8/96 (8.3%)	62/1,764 (3.5%)	2.71 (1.25-5.85)	0.011*	0.76 (0.27-2.14)	0.602	0.77 (0.26-2.30)	0.635
Severe-COPD	9/96 (9.4%)	43/1,764 (2.4%)	4.40 (2.07-9.33)	<0.001*	3.91 (1.36-11.24)	0.011*	4.03 (1.36-11.98)	0.012*
**Non-hypertension (n = 2,878)**								
**Asthma**								
Non-asthma	34/40 (85.0%)	2,628/2,838 (92.6%)	1		1		1	
Mild-asthma	6/40 (15.0%)	188/2,838 (6.6%)	2.47 (1.02-5.95)	0.044	0.70 (0.22-2.23)	0.541	0.60 (0.18-2.04)	0.415
Severe-asthma	0/40 (0.0%)	22/2,838 (0.8%)	N/A		N/A		N/A	
**COPD**								
Non-COPD	36/40 (90.0%)	2,748/2,838 (96.8%)	1		1		1	
Mild-COPD	2/40 (5.0%)	53/2,838 (1.9%)	2.88 (0.68-12.28)	0.153	1.25 (0.19-8.14)	0.814	1.57 (0.22-11.03)	0.652
Severe-COPD	2/40 (5.0%)	37/2,838 (1.3%)	4.13 (0.96-17.77)	0.057	0.90 (0.13-6.27)	0.916	2.20 (0.29-16.81)	0.449
**Hypertension (n = 1,188)**								
**Asthma**								
Non-asthma	74/92 (80.4%)	968/1,096 (88.3%)	1		1		1	
Mild-asthma	14/92 (15.2%)	114/1,096 (10.4%)	1.61 (0.88-2.94)	0.124	1.01 (0.49-2.08)	0.972	0.93 (0.43-1.99)	0.846

Severe-asthma	4/92 (4.3%)	14/1,096 (1.3%)	3.74 (1.20-11.64)	0.023*	2.09 (0.45-9.65)	0.346	1.79 (0.33-9.65)	0.498
**COPD**								
Non-COPD	77/92 (83.7%)	1,041/1,096 (95.0%)	1		1		1	
Mild-COPD	7/92 (7.6%)	39/1,096 (3.6%)	2.43 (1.05-5.61)	0.038*	0.95 (0.34-2.70)	0.930	0.86 (0.27-2.73)	0.803
Severe-COPD	8/92 (8.7%)	16/1,096 (1.5%)	6.76 (2.81-16.29)	<0.001*	4.19 (1.39-12.58)	0.011*	4.04 (1.29-12.59)	0.016*

* Unconditional logistic regression model, Significance at P < 0.05

† Model 1 was adjusted for age, sex, income, obesity, smoking, alcohol consumption, systolic blood pressure, diastolic blood pressure, fasting blood glucose, total cholesterol, CCI scores, number of NSAID used, number of steroid used and hypertension.

‡ Model 2 was adjusted for model 1 plus asthma and COPD.
